# RNA Modifications in Health and Disease

**DOI:** 10.1002/mco2.70341

**Published:** 2025-09-03

**Authors:** Shiqi Li, Ping Luo, Junli Fan, Yirong Li, Jiancheng Tu, Xinghua Long

**Affiliations:** ^1^ Department of Laboratory Medicine Zhongnan Hospital of Wuhan University Wuhan China; ^2^ Department of Clinical Laboratory Medicine Xijing Hospital Fourth Military Medical University (Air Force Medical University) Xi'an China; ^3^ Department of Hematology Zhongnan Hospital of Wuhan University Wuhan China

**Keywords:** cancer, cardiovascular health, m6A methylation, neurodegenerative diseases, PTMs, RNA modifications

## Abstract

RNA modifications, including N6‐methyladenosine (m6A), 5‐methylcytosine, and pseudouridine, serve as pivotal regulators of gene expression with significant implications for human health and disease. These dynamic modifications influence RNA stability, splicing, translation, and interactions, thereby orchestrating critical biological processes such as embryonic development, immune response, and cellular homeostasis. Dysregulation of RNA modifications is closely associated with a variety of pathologies. This review systematically synthesizes recent advances in understanding how dynamic RNA modifications orchestrate health and disease. We critically review the m6A modifications, the most abundant RNA methylation, its association with diseases, and regulations by post translation. We evaluate three interconnected themes: disease mechanisms, where dysregulated m6A drives oncogenesis (e.g., METTL3‐mediated hypermethylation in breast cancer) and contributes to neuropsychiatric/cardiovascular disorders; homeostatic functions, spanning embryogenesis (maternal‐to‐zygotic transition), tissue regeneration (YTHDF1 in muscle), and immune regulation; therapeutic frontiers, including enzyme‐targeting strategies (FTO inhibitors, METTL3 stabilizers) and diagnostic approaches. Our analysis reveals that context‐dependent RNA modification networks operate as biological “switches” whose dysregulation creates pathogenic cascades. We further propose a novel framework for targeting these networks using multiomics integration. This review establishes RNA modifications as central targets for precision medicine, while highlighting critical challenges in clinical translation that demand interdisciplinary collaboration.

## Graphical abstract

Changes in these components profoundly affect the m6A methylation process, leading to a range of physiological and pathological alterations. In recent years, research on the post‐translational modifications of these components has advanced, encompassing ubiquitination, phosphorylation, SUMOylation, O‐GlcNAcylation, and lactylation. This review summarizes the latest findings on the post‐translational modifications of the m6A writer, eraser, and reader. It also examines diseases associated with these m6A modifications. Importantly, the review details the involvement of potential therapeutic targets related to m6A post‐translational modifications. Further investigations into the precise regulation of these modifications will open new avenues to overcome some of the current limitations in cancer therapy.

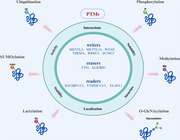

## 1 Introduction

The diversity of RNA modifications has demonstrated itself as a key factor in posttranscriptional gene regulation, occurring widely in various RNA molecules [1]. Over 100 chemical modifications have been identified in cellular RNA, which can affect RNA structure, stability, localization, translation efficiency, and interactions with other molecules [2]. Shortly after the discovery of the classical A, C, G, and U residues of RNA, pseudouridine (Ψ) was first identified in yeast tRNA by Robert Chambers and Robert Shapiro in 1951, which was one of the earliest discovered RNA modifications [3]. In 1965, Holley et al. discovered multiple modified nucleosides (such as dihydrouridine and thiouridine) when sequencing yeast tRNA [4]. In the late 1960s, scientists found dozens of modifications on tRNA, affecting its structure and function. In 1974, Desrosiers et al. discovered N6‐methyladenosine (m6A) at the 5′ end of eukaryotic mRNA. Subsequently, the role of m6A in regulating the fate of both coding RNA (mRNA) and noncoding RNAs (miRNA, tRNA, etc.) has been widely studied [5]. Notably, m6A and other modifications are now recognized as central mediators in disease mechanisms, including cancer progression, neurological disorders, and metabolic syndromes.

RNA modifications, as a crucial layer of epigenetic regulation, have emerged as a central focus in the realm of molecular biology and medicine. These modifications, which encompass a diverse array of chemical alterations to the RNA molecule, play a fundamental role in modulating gene expression, fine‐tuning cellular functions, and maintaining organismal homeostasis. Understanding the complex mechanisms underlying RNA modifications not only provides profound insights into the fundamental principles of gene regulation but also holds great promise for the development of innovative diagnostic and therapeutic strategies. The profound implications of RNA modifications for health and disease drive this rapidly evolving field. Understanding their context‐specific functions not only deciphers fundamental regulatory principles but also unlocks novel diagnostic and therapeutic avenues.

This study specifically investigates how dysregulation of RNA modifications contributes to hepatocellular carcinoma progression/Alzheimer's disease pathogenesis, aiming to identify potential biomarkers and therapeutic targets for triple‐negative breast cancer/Parkinson's disease.

## 2 Types and Mechanisms of RNA Modifications

### 2.1 Common RNA Modifications

RNA modifications encompass a diverse array of chemical alterations that endow RNA molecules with enhanced functional complexity. Among the most prevalent modifications are m6A, 5‐methylcytosine (m5C), N1‐methyladenosine (m1A), N7‐methylguanosine (m7G), 2′‐O‐methylation, Ψ, and so on. Each of these modifications exhibits distinct chemical structures, specific modification sites, and profound impacts on RNA properties [1]. m6A, the most abundant internal modification in eukaryotic mRNA, involves the addition of a methyl group to the N6 position of adenosine residues. It predominantly occurs within the consensus motif RRACH (R = G or A; H = A, C, or U) and is highly enriched near stop codons and in 3′ untranslated regions (3′UTRs). m6A has emerged as a critical regulator of mRNA metabolism, influencing processes such as splicing, export, stability, and translation. For instance, m6A modification within the 3′UTR of certain transcripts can recruit specific RNA‐binding proteins, leading to altered mRNA stability and translational efficiency [6]. m5C, characterized by the methylation of the C5 position of cytosine, is widely distributed across various RNA species, including mRNA, tRNA, and rRNA [7]. In mRNA, m5C sites have been implicated in modulating RNA stability, nuclear export, and translation initiation. Notably, in tRNA, m5C modifications play a pivotal role in maintaining tRNA structure and function, optimizing codon‐anticodon pairing, and regulating translation accuracy [8]. m1A, the methylation of the N1 position of adenosine, is prominently present in tRNA and rRNA. In tRNA, m1A modifications are concentrated in the anticodon loop and adjacent regions, where they contribute to tRNA folding, stability, and decoding efficiency. In rRNA, m1A sites have been shown to influence ribosome assembly and translational fidelity, thereby impacting overall protein synthesis [9]. m7G, involving the methylation of the N7 position of guanosine, is a hallmark modification in the 5′ cap structure of eukaryotic mRNA. This modification is essential for protecting mRNA from exonuclease degradation, facilitating nuclear export, and promoting translation initiation. Additionally, m7G modifications within tRNA and rRNA have been implicated in modulating their structural dynamics and functional properties, further highlighting the versatility of this modification. 2′‐O‐methylation, which occurs at the 2′‐hydroxyl group of ribose sugars, is prevalent in both coding and noncoding RNAs. In rRNA and tRNA, 2′‐O‐methylation is crucial for maintaining RNA secondary and tertiary structures, enhancing stability, and optimizing interactions with ribosomal proteins and other RNA‐binding factors. In mRNA, 2′‐O‐methylation has been associated with modulating splicing patterns, nuclear export, and translational control [10, 11]. Ψ, a structural isomer of uridine, is formed by the enzymatic conversion of uridine residues. Ψ is abundantly present in rRNA, tRNA, and snRNA, where it plays a vital role in RNA folding, stability, and function. In rRNA, Ψ modifications contribute to ribosome biogenesis and translational efficiency, while in tRNA, they influence aminoacylation and decoding accuracy. In snRNA, Ψ modifications are involved in spliceosome assembly and pre‐mRNA splicing, underscoring their significance in RNA processing [12].

**FIGURE 1:**
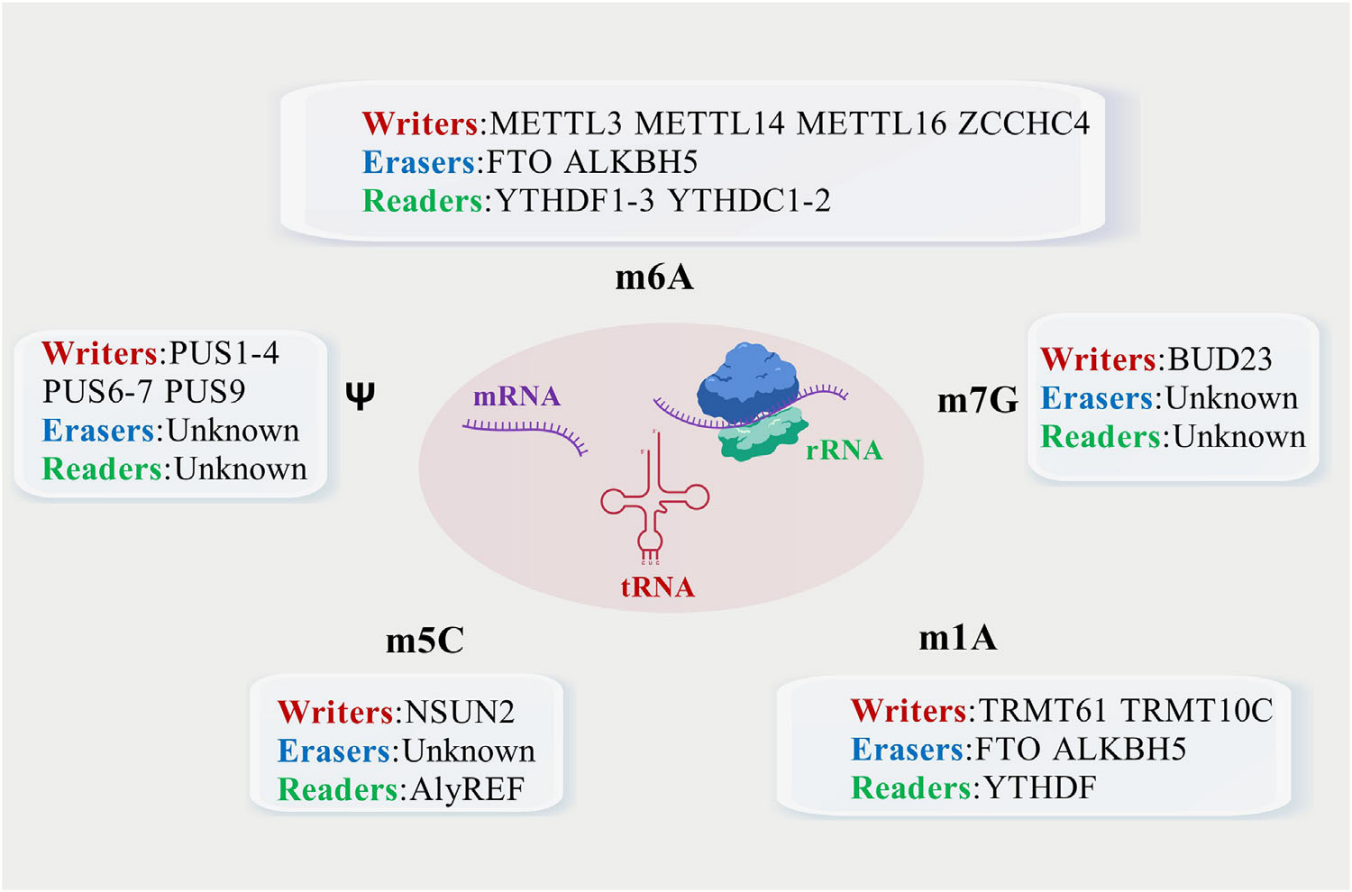
Several common RNAs modifications. Essential RNA chemical marks: m6A (stability/translation), m5C (RNA structure), Ψ (translational fidelity), m1A (RNA protection). Writer/eraser/reader activities shown via color‐coded symbols (red/blue/green). Collectively regulate gene expression outcomes.

These common RNA modifications, through their unique chemical properties and specific modification sites, confer RNA molecules with the ability to respond dynamically to cellular cues, fine‐tune gene expression, and maintain cellular homeostasis. Understanding the precise mechanisms and functional consequences of these modifications is essential for unraveling the complex regulatory networks underlying RNA metabolism and its implications in health and disease (Figure 1).

### 2.2 The “Writer–Eraser–Reader” System

The dynamic regulation of RNA modifications is orchestrated by a sophisticated enzymatic machinery comprising “writers, ” “erasers, ” and “readers.” This system endows RNA modifications with a remarkable degree of plasticity, allowing them to respond rapidly to changing cellular conditions and regulatory demands [1, 13].“Writers” are enzymes responsible for installing specific modifications onto RNA molecules. For m6A, the methyltransferase complex (MTC), which includes methyltransferase‐like protein 3 (METTL3), METTL14, WTAP, and other accessory proteins, catalyzes the transfer of a methyl group from S‐adenosylmethionine (SAM) to the target adenosine residues [14]. METTL3, possessing intrinsic methyltransferase activity, forms a heterodimer with METTL14 to execute the catalytic function, while WTAP and other components play regulatory roles in modulating complex assembly and substrate recognition [15].

“Erasers, ” conversely, are enzymes that reverse RNA modifications, thereby introducing a reversible dimension to the regulatory process. Employing ferrous iron as cofactor and α‐ketoglutarate as cosubstrate, eraser removes m6A, thereby functioning as demethylase [16]. For m6A, two prominent erasers have been identified: fat mass and obesity‐associated protein (FTO) and AlkB homolog 5 (ALKBH5). FTO, belonging to the Fe(II)/α‐ketoglutarate‐dependent dioxygenase superfamily, can mediate the demethylation of m6A and m6Am residues in mRNA and snRNA, as well as m1A residues in tRNA. FTO not only controls mRNA splicing by inhibiting SRSF2 binding at splice sites [17], but also regulates adipogenesis by increasing the relative expression of the pro‐adipogenic RUNX1T1‐S isoform [18]. FTO blocks YTHDF2‐mediated mRNA degradation by reducing m6A levels of cyclin A2 and cyclin‐dependent kinase 2, thereby promoting fat cell cycle progression and adipogenesis. ALKBH5, on the other hand, specifically targets mRNA and oxidatively reverses m6A modifications, contributing to the dynamic regulation of m6A levels [19].

“Readers” are proteins that recognize and bind to modified RNA motifs, translating the modification status into functional outputs [20, 21]. For m6A‐modified RNAs, the YTH domain family proteins, including YTHDF1‐3 and YTHDC1‐2, represent a well‐studied class of readers. YTHDF1 and YTHDF3, through their interaction with the translation machinery, promote protein synthesis, while YTHDF2 recruits RNA degradation enzymes or adaptor proteins, leading to the rapid degradation of target mRNAs [22]. YTHDC1 not only participates in chromatin‐associated RNA decay but also mediates mRNA splicing by recruiting and regulating pre‐mRNA splicing factors [23]. In addition to the YTH domain family, other proteins, such as IGF2BPs, HNRNPs, and eIF3, have also been shown to interact with m6A‐modified RNAs, albeit with distinct functional consequences, highlighting the complexity and versatility of the m6A reader network. IGF2BP can promote RNA expression by enhancing RNA stability. For instance, in one study, it was found that in B‐cell progenitor cells, Lin28b and Igf2bp3 jointly stabilize thousands of mRNAs by binding to the same site, forming an automatic regulatory loop [24, 25]. In colorectal cancer, METTL3, as an oncogene, maintains the stable expression of SOX2 through the m6A IGF2Bp2‐dependent mechanism in CRC cells [26].

The coordinated action of the “writer–eraser–reader” system is exemplified in various cellular processes. For instance, during embryonic development, dynamic changes in m6A modification patterns, mediated by the spatiotemporal regulation of writers, erasers, and readers, have been shown to play a crucial role in cell fate determination and lineage specification [27]. In response to environmental stress, such as hypoxia or nutrient deprivation, alterations in the expression and activity of these enzymes can lead to rapid adjustments in RNA modification profiles, enabling cells to adapt and survive [28]. Dysregulation of this system, whether due to genetic mutations, epigenetic alterations, or environmental perturbations, has been increasingly implicated in a wide range of diseases, including cancer, neurodegenerative disorders, and metabolic syndromes, underscoring its significance as a potential therapeutic target [29].

## 3 Regulation of m6A Modification

Internal modifications of mRNA, such as m6A, significantly influence RNA structure stability and play critical roles in regulating gene expression at the posttranscriptional level. m6A is the most prevalent internal RNA modification in eukaryotic mRNA, among more than 150 identified RNA modifications that are involved in various cellular processes [30]. In addition to m6A, the cap structure at the 5′ end and the poly(A) tail at the 3′ end are also crucial in regulating transcription in eukaryotes. Although m6A modification was initially identified in 1974 [31], it has only recently attracted greater attention with the advancement of high‐throughput sequencing technologies. These advancements have enabled researchers to better understand the role of m6A in a range of biological functions, including RNA metabolism, splicing, translation, and degradation. The m6A modification is mediated by a set of enzymes collectively referred to as m6A “writers, ” “erasers, ” and “readers, ” based on their specific functions. The MTC, often termed the “writer, ” is responsible for catalyzing m6A methylation. This complex includes the catalytic subunit METTL3 and auxiliary subunits such as METTL14, WTAP, VIRMA, RBM15, and ZC3H13 [1]. The stable complex formed by METTL3 and METTL14 plays a key role in substrate recognition [32]. WTAP ensures that the complex is accurately localized to the nuclear speckles and promotes catalytic activity [33]. VIRMA regulates the m6A process by recruiting MTC [34]. ZC3H13 acts as a bridge between WTAP and mRNA binding factor Nito enhance m6A methylation [35]. RBM15 binds the m6A complex as well as recruits it to specific RNA regions [36]. On the other hand, the removal of m6A is catalyzed by demethylases, also known as “erasers.” These include FTO and ALKBH5, which use ferrous iron as a cofactor and α‐ketoglutarate as a cosubstrate to demethylate m6A‐modified RNA [16]. The “reader” proteins, such as IGF2BP1/2/3, YTHDF1/2/3, and ELAVL1, recognize and bind to m6A‐modified RNA to mediate its downstream effects. Together, the writers, erasers, and readers regulate nearly every aspect of RNA metabolism, including splicing, export, folding, translation, and degradation.

**FIGURE 2:**
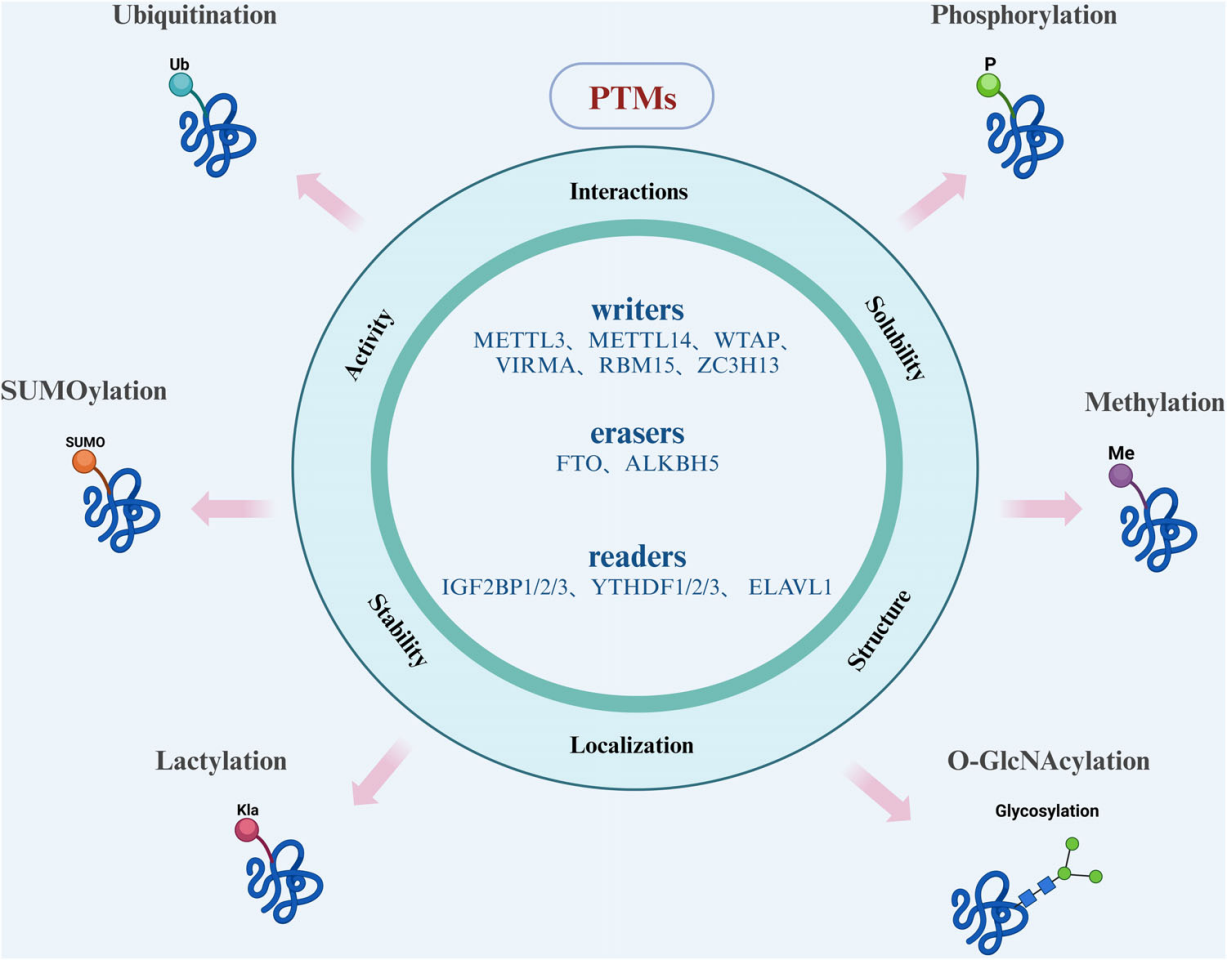
PTMs in RNA methylation. Phosphorylation (METTL3↑/FTO↓), acetylation (ALKBH5 stability), ubiquitination (WTAP degradation). Collectively tune methylation flux via signal‐responsive control.

Given that the synthesis of m6A occurs in the nucleus, m6A methylation on mRNA is completed prior to its cytoplasmic export [37, 38]. This modification has been shown to play a crucial role in various physiological processes, including cell differentiation, immune response, and stress adaptation. Additionally, m6A modification has emerged as an important regulatory mechanism in the pathogenesis of several diseases, particularly cancer. Aberrant m6A modifications are implicated in tumorigenesis by influencing the expression of oncogenes and tumor suppressor genes at the posttranscriptional level, thereby affecting key processes such as cell proliferation, apoptosis, and metastasis. In addition to the intrinsic regulatory properties of m6A methyltransferases, recent studies have demonstrated the critical role of posttranslational modifications (PTMs) in dynamically regulating the activity of these enzymes. PTMs refer to covalent chemical modifications of protein side chains, which can significantly alter protein function. These modifications, including ubiquitination, phosphorylation, SUMOylation, O‐GlcNAcylation (O‐GlcNAc), and lactylation, can modulate the activity of m6A writers and influence RNA metabolism [39]. These modifications often trigger unique protein–protein interactions (PPIs), such as direct binding of modified amino acid residues to read proteins, resulting in downstream functional outcomes. Abnormal PTM processes have been implicated in the progression of cancers [40], viral infections [41], and cardiovascular diseases (CVDs) [42] (Figure 2).

### 3.1 Regulation of Ubiquitination on m6A Writers

Protein ubiquitination, one of the most common PTMs, mainly controls protein degradation and homeostasis [43]. Ubiquitination is the process by which ubiquitin (Ub) molecules organize the proteins in cells under the control of a number of unique enzymes, pick out the target protein molecules from among them, and modify the target protein in a particular way. A sequence of events involving the Ub‐activating enzyme E1, the Ub‐binding enzyme E2, and the Ub ligase E3 take place during ubiquitination modification. Among them, highly specific Ub ligases (E3) play a crucial role in the ubiquitination of target proteins. Protein location, metabolism, function, regulation, and destruction are all significantly influenced by ubiquitination. The 26S proteasome then degrades the ubiquitinated protein when the E3 Ub ligase transfers the Ub molecule from the E2 enzyme to substrate proteins. Recently, more and more scientists have found that ubiquitination plays an important role in the regulation of m6A writers.

#### 3.1.1 Ubiquitination of METTL14

METTL3 and METTL14 complexes play crucial roles in catalytic m6A methylation. Therefore, the stability of the METTL14 protein is closely linked to m6A methylation. Recent studies have demonstrated that the competitive interaction between METTL3 and the E3 ligase STUB1 regulates the stability of METTL14 [44].

STUB1 mediates the ubiquitination‐dependent degradation of METTL14 by directly interacting with it to facilitate the ubiquitination of lysine residues K148, K156, and K162. This series of events results in a significant decrease in overall m6A levels. STUB1 (STIP1 homologous and U‐box protein 1) acts as an E3 Ub ligase that specifically targets and ubiquitinates METTL14, leading to its proteasomal degradation. Conversely, METTL3 has a stabilizing effect on METTL14 at the protein level [45, 46]. METTL3 exerts a protective role by preventing METTL14 degradation, thereby maintaining the dynamic balance of m6A. The mechanism involves METTL3 directly binding to the ubiquitination domain of METTL14, protecting its ubiquitination sites from STUB1‐induced degradation. Furthermore, pan‐cancer analysis based on the TCGA database revealed a strong negative correlation between METTL14 and STUB1 expression across most cancers, particularly in cholangiocarcinoma (CCA) [47]. High levels of STUB1 expression may hinder tumorigenesis progression by promoting METTL14 degradation in vivo. This suggests that STUB1 and METTL14 may have potential utility as diagnostic markers for CCA [44].

Unfolded or misfolded proteins accumulate in endoplasmic reticulum lumen, triggering a stress‐adapted unfolded protein response (UPR). METTL14 is involved in the adaptive response of UPR. The misfolded protein competitively binds to HRD1–ERAD, which is the enzyme that ubiquitinates METTL14. This leads to reduced ubiquitination of METTL14, stable expression of METTL14, and thus methylation by binding to the downstream CHOP gene that mediates apoptosis. Promote the degradation of CHOP gene and reduce the pressure of cell injury and apoptosis [48]. This mechanism may influence the prognosis and progression of liver injury caused by alpha‐1 antitrypsin deficiency (AA‐TD).

The study reveals a significant role of miR‐99a‐5p in esophageal squamous cell carcinoma (ESCC), where its expression is markedly reduced. miR‐99a‐5p was found to inhibit cancer stem cell (CSC) persistence and the radioresistance of ESCC cells, and its downregulation was associated with an unfavorable prognosis in ESCC patients. Mechanistically, a METTL14–miR‐99a‐5p–TRIB2 positive feedback loop was uncovered, enhancing CSC properties and promoting radioresistance in ESCC. METTL14, an m6A RNA methyltransferase, is downregulated in ESCC and plays a crucial role in suppressing TRIB2 expression. This suppression occurs via miR‐99a‐5p, which degrades TRIB2 mRNA by targeting its 3′UTR. Conversely, TRIB2 promotes the Ub‐mediated proteasomal degradation of METTL14 in a COP1‐dependent manner, forming a reciprocal regulatory circuit. METTL14 also enhances miR‐99a‐5p levels by modulating m6A‐mediated processing of pri‐mir‐99a through DiGeorge critical region 8 (DGCR8). The hyperactivation of TRIB2 resulting from this positive feedback loop was strongly associated with increased radioresistance and CSC‐like traits in ESCC cells. Additionally, TRIB2 was shown to activate HDAC2, which in turn epigenetically represses p21 via the activation of the Akt/mTOR/S6K1 signaling pathway. Importantly, pharmacologic inhibition of HDAC2 effectively mitigated the TRIB2‐mediated effects both in vitro and in patient‐derived xenograft models. These findings highlight the critical role of the METTL14–miR‐99a‐5p–TRIB2 feedback loop in promoting the aggressive and radioresistant phenotype of ESCC, providing insight into potential therapeutic targets. In particular, TRIB2 and HDAC2 emerge as promising targets for disrupting this feedback loop, offering a potential strategy to overcome CSC‐related radioresistance in ESCC. The pharmacologic inhibition of HDAC2 could serve as a viable therapeutic approach for ESCC patients exhibiting hyperactivation of the METTL14–miR‐99a‐5p–TRIB2 axis. [49].

Ub ligase E3 and the more than a hundred deubiquitinating enzymes (DUBs, which have the opposite function) in the human body coordinate Ub signaling by providing selectivity for different substrates. The balance between ubiquitination and deubiquitination is finely regulated to ensure proper protein homeostasis and response to cellular stimuli and stressors. Currently, there are seven known families of DUBs, six of which contain different cysteine (Cys) protease folds (USP family, Josephin/MJD family, OTU family, UCH family, ZUFSP family, and MINDY family), while one family contains a zinc metalloprotease fold (JAMM family). [50]. The indirect role of DUBs in regulating oncogenes may be widespread. DUBs can mediate the regulation of E3 ligase complexes or acetone‐induced signaling cascades, leading to a global imbalance in the Ub proteome within cells, affecting many ubiquitinated proteins rather than just a specific target. Moreover, DUBs can also influence translation and transcription because ubiquitination affects gene expression at different levels, such as due to epigenetic dysregulation or dysregulated transcriptional activation of oncogenes [51].

#### 3.1.2 Ubiquitination of METTL3

T Cardiac function and protein homeostasis are two important aspects of Ub–proteasome system maintenance. In primary neonatal rat cardiomyocytes and Ang II‐induced hypertrophic heart, it has been discovered that the deubiquitinating enzyme Ub‐specific proteolytic enzyme 12 (USP12) is elevated. Furthermore, it exacerbates Ang II‐induced cardiac hypertrophy by upregulating the expression of METTL3. According to the mechanism, USP12 binding inhibits the degradation of p300, a methyltransferase that facilitates H3K27 acetylation in the METTL3 promoter, stabilizing it and triggering transcription of its downstream gene, METTL3. It is interesting to note that USP12 mediates a stabilizing impact on METTL3 via p303 rather than acting directly on it. Antagonizing p300 may be a potent therapy for cardiac hypertrophy as elevation of p300 in response to pro‐hypertrophic and pro‐fibrotic stress signals is linked to increased expression of hypertrophic and fibrotic genes [52].

It has been shown that the ERK3 pathway and the deubiquitinating enzyme USP5 work in concert to influence the stability of the MTC and, therefore, the amount of m6A, which is crucial for both pathology and physiology in vivo. Mechanistically, ERK phosphorylates METTL3 by interacting with it and WTAP; meanwhile, ERK activation deubiquitinates METTL3 by translocating USP5 to the nucleus, where it interacts with phosphorylated METTL3. Composed of five different domains, the large protein known as USP5 is composed of the cryptic ZnF domain, the ZnF domain, the C‐box domain, the UBA1/UBA2 domain, and the H‐box domain [53]. With METTL3, the cryptic ZnF domain has the best binding capacity. USP5 may cleave the polyubiquitin bonds K6, K11, K29, K48, and K63. METTL3 must be deubiquitinated by the action of ERK, which phosphorylates both WTAP and METTL3 at S306 and S341 [54].

In ovarian cancer and other malignancies, low expression of phospholipase A2 activating protein (PLAA) is linked to a poor prognosis [55]. Another function of PLAA is to regulate protein ubiquitination [56]. According to research, PLAA has no effect on the mRNA level of METTL3, but it does influence the protein level. METTL3 ubiquitination is decreased along with PLAA downregulation, but the further mechanism is unknown [57]. Reduced ubiquitination of METTL3 leads to stable expression and elevated levels, which lead to elevated methylation levels of TRPC3. TRPC3, a member of the TRP channel superfamily, is essential for maintaining calcium concentration levels [58]. The proliferation, invasion, and migration of tumors are significantly influenced by TRPC3 [59].

By interacting with METTL3, peptidyl‐prolyl cis–trans isomerase NIMA‐interacting 1 (PIN1) inhibits ubiquitination and lysosome degradation. It increases the modification of m6A transcriptional coactivators with PDZ‐binding motifs (TAZ) and epidermal growth factor receptor mRNA, leading to its effective translation, by stabilizing METTL3, which in turn stabilizes METTL3. According to certain research, PIN1 regulates the translation of mRNA, and the PIN1/METTL3 axis may serve as an alternative therapeutic target for breast cancer.

Despite increasing evidence of the importance of m6A methylation modification in tumor progression and a rationale for targeting m6A methylation modifiers in cancer therapy, only a few epigenetically targeted drugs have entered clinical trials. METTL3 is an ideal therapeutic target. Studies have shown that METTL3 plays a positive role in the metastasis and spread of ESCC. EGR1 is a key downstream target of METTL3 in promoting ESCC metastasis and plays an oncogenic role in an m6A‐dependent manner. The interaction between METTL3 and STUB1 promotes its ubiquitination and degradation, which is carried out through the E3 ligase of STUB1. Amino acids Lys‐480 and Lys‐459 are the key ubiquitination sites of METTL3. In this study, the potential of elvitegravir, an United States Food and Drug Administration‐approved drug for the treatment of human immunodeficiency virus (HIV) infection, targeting METTL3 in ESCC was highlighted [60].

#### 3.1.3 Ubiquitination of WTAP

Ub‐mediated methyltransferase WTAP is associated with the expression of prostaglandin receptor 3 (EP3). The absence of EP3 accelerates Ub‐mediated WTAP degradation. The brown fat group is a nonshivering thermogenic energy source that maintains body temperature in hibernating animals and infants [61]. Transplantation of BAT can reverse obesity and insulin resistance induced by a high fat diet in rodents. It has been found that prostaglandin receptor signaling is a key promoter for brown adipogenesis to enhance the expression of thermogenic gene programs. The loss of EP3 accelerates the degradation of WTAP, thereby reducing the m6A modification‐dependent stability of zinc finger protein 410(Zfp410) transcription factor (TF) (Zfp410), thereby impacting Browning during BA differentiation and exacerbating diet‐induced obesity [62].

Interferon's harmful effects on the body during viral infection are mediated by WTAP. Type I interferons are crucial for innate protection against viruses. Kind I interferons are produced as a result of viral nucleic acids. Even while interferon‐I serves a crucial defensive function against pathogens, an excessive interferon response contributes to immunological pathogenesis by excessively stimulating inflammation, such as in infectious and inflammatory autoimmune disorders. It has been shown that WTAP is depleted during viral infection by ubiquitination–proteasome‐mediated destruction. As a consequence, IRF3 and IFNAR1 mRNAs have less m6A methylation, which inhibits their capacity to translate, and their mRNAs become unstable. In order to prevent the host from overreacting to the immunopathologic consequences of damaging interferon‐I, careful regulation of IRF3 and IRNAR1 by m6A alteration is necessary.

### 3.2 Regulation of Ubiquitination on m6A Pathway Erasers

#### 3.2.1 Ubiquitination of FTO

Only two m6A demethylases, FTO and ALKBH5, have been discovered so far in mice and humans. A member of the AlkB family of ‐ketoglutarate‐dependent dioxygenase proteins, FTO is a nuclear protein [63]. FTO plays a significant role in the control of cell growth and translation [64, 65] since it is highly expressed in the nucleus, where demethylation takes place, and in the cytoplasm, where it may function as a “amino acid sensor” and target the mTORC1 signaling pathway [63]. The nuclear localization and degradation of FTO may be controlled by its ubiquitination. The primary ubiquitination site responsible for FTO breakdown is Lys‐216 [66].

An m6A demethylase known as the FTO was first discovered as a gene linked to both obesity and energy metabolism. It is the first demethylase discovered [67]. It is dysregulated in many malignancies and is crucial for the growth of tumors, the self‐renewal of tumor stem cells (CSC), and the control of the microenvironment. FTO has variable effects on tumor growth and prognosis in various malignancies, acting as a carcinogen in cases of breast cancer, cervical cancer, and melanoma [68, 69, 70]. In ovarian, hepatocellular, and renal malignancies, the incidence was negatively correlated with tumor advancement. Recent research has shown that FTO acts as a preventative in colorectal cancer. FTO, also known as alpha‐ketoglutarate‐dependent dioxygenase FTO, is an enzyme that needs oxygen to operate. Hypoxia in the tumor microenvironment, which causes FTO to be degraded by ubiquitination, is linked to a poor prognosis for people with colorectal cancer. Hypoxia was observed to enhance the degree of FTO ubiquitination, with K216 serving as the primary location of FTO ubiquitination in colorectal cancer cells. The downstream gene of FTO is MTA1. Through demethylation, FTO lowers the expression level of MTA1. The insulin‐like growth factor 2 mRNA binding protein (IGF2BP) family member IGF2BP2, whose binding location is congruent with the FTO‐controlled m6A modification site, is the “reader” of MTA1. Additionally, IGF2BP2 decreases MTA1 expression in the absence of FTO, indicating that FTO and IGF2BP2 control MTA1 expression in colorectal cancer via methylation [71].

AMD1 expression is upregulated in HCC tissues, which is a bad prognostic indicator for HCC. Through FTO‐mediated mRNA demethylation, AMD1 dramatically increased the expression of NANOG, SOX2, and KLF4 in HCC cells. The scaffold protein IQGAP1 is modified by high amounts of AMD1, which raises the level of SPD in HCC cells and improves the interaction between IQGAP1 and FTO. This relationship increases FTO protein production by enhancing FTO phosphorylation and decreasing ubiquitination [72].

Ischemic stroke is characterized by neurological deficits resulting from cerebrovascular occlusion [43, 73]. In order to create a new vascular network to supply blood to hypoxic tissue, endothelial cell (EC) performs highly coordinated morphogenetic events during vascular repair, including basement membrane degradation, EC germination and branching, vascular cavity formation, vascular anastomosis and maturation, and parietal cell recruitment [74, 75]. The brain contains large amounts of circular RNAs (circRNAs), of which circSCMH1 regulates m6A methylation to facilitate vascular healing [76]. CircumSCMH1 binds to FTO and promotes nuclear translocation of FTO in EC, controlling the stability of Plpp3mRNA as the mechanism—lysophosphatidic acid's biological activity is regulated by the cell surface enzyme LPP3, which is encoded by Plpp3 [77]. It causes Plpp3 mRNA to become methylated and prevents Plpp3 from being degraded in the EC. CircSCMH1 was discovered to stimulate FTO ubiquitination, boost FTO Ub‐K13 levels, and ease the movement of FTO from the cytoplasm to the nucleus [78]. It has been shown that endothelium‐targeted overexpression of FTO increases vascular area, total length, and branch count, hastening the recovery of motor function after PT stroke.

Hexokinase HK2 promotes metabolic recombination and the development of cancer that is malignant. According to studies, the HPV E6E7 oncogene causes GSK3 to be expressed, which encourages the ubiquitination and proteasome degradation of FTO and lowers the level of FTO protein. This prevents HK2 mRNA from maturing and translating while keeping the pre‐HK2 mRNA in the nucleus [79]. Additionally, GSK3 has been shown to have this effect in colorectal cancer, where it prevents the development and spread of CRC by activating the MZF1/c‐Myc axis via FTO [80]. GSK3 stimulates FTO phosphorylation during myocardial ischemia/reperfusion (MI/R) damage, which increases FTO ubiquitination and degradation. Myocardial apoptosis and increased oxidative stress may result from a reduction in FTO because it can suppress the expression of the gene Myc [81]. Studies on the effects of FTO in cervical cancer, however, are inconsistent and hotly debated. The amount of FTO mRNA in cervical cancer tissue (*N* = 304) was lower than that in normal cervical tissue (*N* = 3), according to one research based on data analysis of cervical cancer tissue samples in TCGA [82]. Another research that has been mentioned came to the conclusion that FTO transcripts were strongly expressed in cervical cancer tissues (*N* = 20) compared with normal cervical tissues (*N* = 50) based on its own examination of clinical tissue samples that were obtained [83]. FTO knockdown inhibited the proliferation and migration of HeLa and SiHa cells [70], while overexpression of FTO promoted chemical resistance of cervical cancer cells [84]. In BLCA tissues and cell lines, increased protein levels of FTO are generated by posttranslational deubiquitination induced by Ub‐specific peptidase 18 in the N‐terminal protein domain as opposed to high mRNA expression. Stable FTO promotes BLCA cell proliferation and migration in vitro and tumor development in vivo by decreasing the m6A methylation of pyrrolidine 5‐carboxylate reductase 1 (PYCR1) and lengthening the mRNA half‐life of PYCR1 [85].

#### 3.2.2 Ubiquitination of ALKBH5

It is a demethylase, ALKBH5. ALKBH5 is a nonheme Fe (II)/‐KG‐dependent dioxygenase that is a member of the conserved AlkB family that facilitates N‐alkylated nuclear base repair by oxidative demethylation [86, 87]. It influences RNA output, nucleus metabolism, gene expression, and is mostly expressed in the testes. It has been demonstrated to have an impact on mouse fertility [19]. Demethylation caused by ALKBH5 controls gene expression by affecting a number of RNA metabolism activities, including pre‐mRNA processing, mRNA degradation, and translation pathways.

It was found that ROS specifically promoted the SUMOylation of ALKBH5 through the activation of ERK/c‐Jun N‐terminal kinase (JNK) signaling pathway but not the SUMOylation of FTO, METTL3, and METTL14. METTL14 enhanced the interaction between ALKBH5 and UBC9. Inhibiting the binding of ALKBH5 and SENP1 to achieve SUMOylation [39]. Mechanically, phosphorylation of ALKBH5 enhances the interaction between ALKBH5 and the small Ub‐like modifier (SUMO) E2 binding enzyme UBC9, triggering the SUMOylation of ALKBH5, leading to the inhibition of m6A demethylase activity and the overall increase of m6A methylation.

As a possible ALKBH5 deubiquitination enzyme, Ub‐specific peptidase 36 (USP36) is essential for ALKBH5 stability and AlkBH5‐mediated gene expression regulation in glioblastoma (GBM). USP36 depletion severely inhibits cell division, affects GSCS self‐renewal, and renders GSCS susceptible to temozolomide treatment [88].

### 3.3 Regulation of Ubiquitination on m6A Pathway Readers

#### 3.3.1 Ubiquitination of YTHDF1

In the context of YTHDF1, it has been noted that reports on its PTMs are scarce. However, recent studies have begun to shed light on its regulation, particularly through ubiquitination processes. A 2023 study revealed that the deubiquitinase USP47 plays a critical role in protecting YTHDF1 from ubiquitination, thereby promoting c‐Myc translation. This process is crucial for maintaining regulatory T cell (Treg) metabolic and functional homeostasis. USP47 prevents the association of YTHDF1 with the translation initiation machinery, reducing m6A‐mediated translation efficiency of c‐Myc. Consequently, this regulatory mechanism is essential for Treg homeostasis, and its dysregulation can result in immune disorders and hyperglycolysis due to excessive c‐Myc activity. Furthermore, USP47 was found to correlate with tumor‐infiltrating Tregs in colorectal and gastric cancer, indicating its potential relevance in the tumor microenvironment and immune modulation.

This new understanding of USP47‐mediated deubiquitination highlights an important layer of control in the regulation of YTHDF1 and its downstream effects, including translation and metabolic processes. The inclusion of this regulatory axis expands upon our understanding of the role of YTHDF1 in PTMs and emphasizes the complexity of its involvement in cellular functions such as immune regulation and tumor progression [89]. Studies have found that curcumin can reduce the expression of ALKHB5, resulting in increased methylation level of TNF receptor‐related factor 4 (TRAF4) mRNA. TRAF4 mRNA methylation is recognized and constrained by YTHDF1, thus enhancing the translation of TRAF4. TRAF4 acts as an E3 cycloubiquitin ligase. The Ub–proteasome pathway promotes the degradation of PPARγ, a regulator of adipocyte differentiation, thereby inhibiting lipogenesis [90]. In sepsis, overexpressed YTHDF1 inhibits CLP‐induced inflammation in mice by upregulating E1 Ub protein ligase 1 to promote NLRP3 ubiquitination and inhibit caspase 1‐dependent pyroptosis, thereby alleviating sepsis [91].

#### 3.3.2 Ubiquitination of YTHDF2

By destroying numerous cancer proteins in tumors, the tumor suppressor FBW7 preserves genomic integrity and inhibits the growth and development of cancers. According to studies, the m6A binding protein YTHDF2 promotes the growth of ovarian cancer cells, and the tumor‐inhibitory effect of the latter is dependent on FBW2‐induced protein hydrolysis of YTHDF2. Ovarian cancer cells undergo apoptosis as a result of FBW7's mechanism, which decreases YthDF2‐mediated methylation and enhances the stability of pro‐apoptotic BMF mRNA [92].

According to research, YTHDF2 is also involved in controlling the cell cycle, and its absence might delay the start of mitosis. The mechanism is that CDK1 is inhibited by WEE1 via phosphorylation, whereas YTHDF2 encourages WEE1 transcriptional decrease. By raising the fraction of the inactive CDK1 form CDK1–Y15, excessive WEE1 protein accumulation in the absence of YTHDF2 delays the transition from the G2 phase to the M phase. Mitosis is controlled by phosphorylation and dephosphorylation of regulatory proteins including CDK1 and WEE1. The stability of the YTHDF2 protein is reliant on CDK1 activity. The CRLNEDD8 pathway, which is known to control a variety of physiological functions, including cell cycle progression, mediates the degradation of the YTHDF2 protein in the proteasome [93]. CDK1 may maintain the stability of YTHDF2 by preventing the association between YTHDF2 and the E3 ligase complex associated with Skp2. This indicates that the feedforward regulatory ring composed of CDK1, YTHDF2, and WEE1 regulates mitotic entry [94]. CDK1 inhibitors have great potential in inducing degradation and apoptosis of YTHDF2 in acute myeloid leukemia (AML). The loss of YTHDF2 leads to apoptosis of AML cells, which makes YTHDF2 a potential target for the treatment of AML [95].

#### 3.3.3 Ubiquitination of YTHDF3

Patients with HCC often have a bad prognosis due to the greatly upregulated expression of YTHDF3 in cancerous tissues. Tumor invasion, migration, and proliferation are all aided by YTHDF3. By encouraging the production of phosphofructose kinase PFKL at the mRNA and protein levels, YTHDF3 mechanically stimulates aerobic glycolysis. Through EFTUD3, a central splice body component involved in pre‐mRNA splicing, PFKL interacts with YTHDF2. By preventing EFTUD3 from ubiquitinating YTHDF3 protein, PFKL may positively regulate the expression of YTHDF2 protein. This suggests that YTHDF3 and PFKL work together to produce a positive feedback loop that controls the metabolism of cancer cells in hepatocellular carcinoma [96].

#### 3.3.4 Ubiquitination of IGF2BP1

Hepatocellular carcinoma, among other malignancies, expresses the carcinogenic protein insulin‐like growth factor 2 mRNA binding protein 1 (IGF2BP1), and FBXO45 is an E3 Ub ligase. By mechanically promoting IGF2BP1 ubiquitination and subsequent activation at Lys2 and Lys1, FBXO45 induces liver carcinogenesis in vivo and in vitro by upregulating PLK1 expression, inducing cell proliferation, and ubiquitination of IGF2BP1. IGF2BP1 activation and ubiquitination, as well as the consequent overexpression of PLK1, are caused by FBXO45 and contribute significantly to the development of liver tumors [97].

#### 3.3.5 Ubiquitination of IGF2BP2

circRNAs were first discovered more than 40 years ago [98]. Furthermore a significant number of circRNA have been discovered in eukaryotes thanks to developments in high‐throughput RNA sequencing and circRNA‐specific bioinformatics methods, some of which are linked to tumor growth. In order to decrease the stability of IGF2BPs, circNDUFB25 may create a terpolymer complex with TRIM2, circNDUFB25, and IGF2BPs. The mechanism is that circNDUFB2 functions as a scaffold to encourage TRIM25‐mediated ubiquitination of IGF2BPs. The RNA binding activity of TRIM25 is necessary for its Ub ligase function. The efficient generalization of IGF25BP by TRIM2 depends on circNDUFB2 [99]. CircumEZH2 expression was markedly upregulated in colorectal cancer tissues, and it was associated with the clinical characteristics of colorectal cancer patients. In vitro and in vivo CRC cell proliferation and migration were markedly reduced when circEZH2 was knocked down. The m6A reader IGF2BP2 and circEZH2 interact, preventing the reader's ubiquitination‐dependent destruction. IGF2BP2 is upregulated in CRC in response to miR‐133b downregulation, which may be caused by the circEZH2 sponge—miRNA sponges are the main mechanism by which circRNAs exercise their biological effects [100]. Additionally, via altering CREB1 expression, circEZH2/IGF2BP2 speeds up the course of CRC and improves CREB1 mRNA stability in the disease. Through ERK2/1, PKA, PKC, or CaMKII signaling pathways, which are involved in cell proliferation, differentiation, apoptosis, neovascularization, inflammatory response, and cancer, CREB1 promotes the expression of proto‐oncogenes such cyclin A2, EGR‐9, MMP3/1, GSK2A, and noncoding RNA [101, 102, 103, 104, 105].

LncRNAs play a critical function in influencing the course of tumors in a complicated regulatory network. A high expression of the carcinogenic lncRNA LIRIS is linked to a poor outcome in CRC patients. IGF2BP2's ubiquitination site is where LIRINRIS interacts. Additionally, this binding stabilizes downstream mRNA, including MYC mRNA, inhibits IGF2BP2 from degrading through the ubiquitination–autophagy pathway, and the downregulation of MYC‐related metabolic enzymes results in decreased glycolysis [106].

#### 3.3.6 Ubiquitination of IGF2BP3

CircNEIL3 has been discovered to accelerate the development of gliomas and tumors by maintaining the cancer‐causing protein IGF2BP3 and preventing HECTD4 from mediating ubiquitination. A member of the HECD family of E3 Ub ligases, HECTD4 connects target proteins to polyubiquitin to encourage ubiquitination [107, 108]. Similar to how circNEIL3 interacts with IGF2BP3, the HECTD4 binds to the KH2‐3 domain of IGF2BP3. CircNEIL3 prevented IGF2BP3 from being ubiquitinated by preventing IGF2BP3 and HECTD4 from interacting through spatial bits [109].

IGF2BP3 is a key biomarker for systemic malignancies and has a strong correlation with many different forms of cancer. IGF2BP3 is regarded as a marker for neuroblastoma with high clinical importance [110]. Increased IGF2 protein and the activation of the PI3K and MAPK signaling pathways are results of IGF2BP3 overexpression. resulting in cell invasion, proliferation, and transformation [111].

IGF2BP3's E3 Ub ligase is called MKRN2. Exogenous MKRN2 has the ability to ubiquitinate overexpressed IGF2BP3, and the RING domain of this protein is essential for IGF2BP3 ubiquitination. IGF2BP3 is ubiquitized at several locations by MKRN2. Important IGF2BP3 downstream targets include CD44 and PDPN. In other words, overexpression of MKRN2 downregulates the mRNA levels of PDPN and CD44, while overexpression of IGF2BP3 upregulates the expression of these two genes. MKRN2 controls the expression of CD3 and PDPN in an IGF44BP2‐dependent way. Low PDPN and CD44 levels in patients with central nervous system malignancies were associated with prolonged overall survival [112].

LncRNA MNX1–AS1, an antisense RNA of MNX1, has been found to have a carcinogenic function in many cancer types via a number of ways. MnX1–AS1 has been shown to be overexpressed in all cancers in every study that is currently accessible, and it is strongly related to clinicopathological characteristics. include the size of the tumor, the clinical stage, the overall survival, and so on [113, 114, 115]. MNX1–AS1 prevents the proteasome from causing IGF2BP3's ubiquitination and degradation, stabilizing the protein at the molecular level. The theory is that through improving the connection between USP2 and IGF2BP3, MNX1–AS1 shields IGF2BP3 from Ub–proteasome‐dependent degradation [116]. USP2 also maintains Hippo signaling inactivation via the IGF3BP4 / TEAD7 positive feedback loop.

### 3.4 Regulation of Phosphorylation on m6A Writers

Phosphorylation is a component of common PTM. A protein or an intermediary metabolite gets phosphorylated when a phosphoric acid group is added. Phosphatases are the name for enzymes that dissolve phosphate groups. The majority of amino acids (the building blocks of proteins) that undergo protein phosphorylation are serine, with threonine coming in second.

#### 3.4.1 Phosphorylation of METTL14

METTL3, METTL14, and WTAP have all been discovered to be phosphorylated by alphaherpesvirus kinases. This phosphorylation results in the inactivation of the complex and an almost total loss of m6A levels in the mRNA of virus‐infected cells. The m6A MTC must be phosphorylated and inhibited by viral US3 protein production, although its kinase activity is not required. The m6A writing complex dissociates from chromatin during alphaherpesvirus infection but maintains its integrity in the nucleus, indicating that it may no longer bind to nascent mRNA in cells infected with alphaherpesvirus [117]. The inhibition of the m6A MTC depends on US3.

#### 3.4.2 Phosphorylation of METTL3

Studies have found that the activation of ERK pathway promotes the m6A methylation of mRNA, and ERK interacts with METTL3 through phosphorylation. Following MEK stimulation, active ERK either forms dimers to activate the cytoplasmic substrate or translocates to the nucleus to activate the nuclear substrate [118]. ERK's nuclear substrate is METTL3 complex. When phosphorylated at the serine/threonine‐proline (S/T‐P) motif, ERK exhibits selectivity. METTL3 is phosphorylated at S3/S43/S50 by ERK. The activity of the RNA m6A MTC is increased by ERK‐induced phosphorylation, and USP5 is necessary for ERK‐mediated stabilization of METTL3. USP5, an enzyme that inhibits protein ubiquitination, stabilizes METTL3 by deubiquitinating it as the mechanism [119]. ERK‐dependent METTL3 phosphorylation promotes mESC differentiation.

The most dangerous kind of DNA damage is double‐strand breaks (DSBS), which, if left unrepaired, may result in genomic instability or cell death. A normal human cell is thought to collect roughly 105 spontaneous DNA damage every day [120]. Homologous recombination (HR)‐mediated DSBR and nonhomologous terminal junction (NHEJ)‐mediated DSBR are the two primary DSB repair processes in mammalian cells. In contrast to HR‐mediated DSBRS, which needs homologous DNA templates and produces high fidelity DSBRS, NHEJ may be carried out without homologous DNA templates and is consequently regarded as mutagenic [121]. This pathway requires a well‐regulated cellular signaling pathway to coordinate the actions of sensors, transducers, and effectors [122]. Human cancers and age‐related illnesses are linked to HR‐mediated abnormalities in DSBR and NHEJ [123]. It has been discovered that ATM, the master regulator of DDR, phosphorylates and activates METTL3. Its downstream effector encourages DNA repair while slowing down or stopping cell cycle development. The alteration of the m6A RNA on the DSB is catalyzed by activated METTL3. Additionally, the m6A‐modified RNA is protected by the m6A reader protein YTHDC1, which also promotes the buildup of DNA–RNA hybrids on DSBS. This METTL3–m6A–YTHDC1 route could be a crucial one for increasing DSBR effectiveness in healthy cells. Therefore, the researchers propose that blocking this pathway could increase the efficiency of radioactive and chemotherapy‐based cancer treatments [124].

The transition from nonalcoholic fatty liver disease (NAFL) to cirrhosis requires the presence of nonalcoholic steatohepatitis (NASH). According to studies, METTL3 has a significant negative regulatory role in the development of NASH. Through enhanced CD36‐mediated hepatic free fatty acid absorption and CCl2‐induced inflammation because of increased chromatin accessibility in the Cd36 and Ccl2 promoter area, Mettl3‐specific deletion in hepatocytes promotes the development of NAFL to NASH. The mechanism is that METTL3 binds to the Cd36 and Ccl2 promoters directly and then recruits HDAC1/2 to cause the deacetylation of H3K9 and H3K27 in these promoters, preventing Cd36 and Ccl2 from being transcribed. In NASH, METTL3 is moved from the nucleus to the cytoplasm, which is connected to METTL3 phosphorylation by CDK9 [125]. Reduced nuclear METTL3 helped make the change from NAFL to NASH possible. While CDK3‐mediated phosphorylation at other METTL9 sites may prevent nuclear localization, certain METTL3 phosphorylation sites may not result in nuclear/cytoplasmic translocation 38. The m6A alteration of mRNA is crucial for antiviral immunity. The essential kinase TBK1 phosphorylates the fundamental transferase METTL3 as part of the antiviral pathway. By interacting with the translation complexes necessary to improve protein translation, phosphorylated METTL3 encourages antiviral responses [126].

#### 3.4.3 Phosphorylation of WTAP

As previously indicated, it has been discovered that ERK phosphorylates WTAP at S306/S341 and METTL525 at S3/S43/S50. Following deubiquitination by USP5, this stabilizes the MTC and accelerates the growth of the tumor [119].

Solid tumors have a significant amount of neutrophils. It has been discovered that C5ar1‐positive neutrophils, a group of neutrophils linked to the growth of breast cancer, increase BC cell glycolysis via upregulating ENO1 expression. The process is that C5AR1‐positive neutrophils release IL1 and TNF‐, which together stimulate extracellular regulatory protein kinase 1/2 (ERK1/2) signaling, phosphorylate serine at position 341 of WTAP protein, stabilizing it. The stability of WTAP also altered the glycolysis activity of BC cells and further encouraged the methylation of RNA m6A in ENO6. WTAP silencing reverses the influence that C5ar1‐positive neutrophils have on breast cancer development in vivo [127]. The possibility of using C5AR1‐positive neutrophils as therapeutic targets for breast cancer is supported by this study. As previously indicated, it has been discovered that alpha herpes virus kinase causes the phosphorylation of METTL3, METTL14, and WTAP, among other members of the m6A MTC. This is connected to the complex's suppression and a nearly total loss of the m6A level in the mRNA of virus‐infected cells [117].

### 3.5 Regulation of Phosphorylation on m6A Erasers

#### 3.5.1 Phosphorylation of FTO

Several phosphorylated amino acids have been identified in FTO [128], and their phosphorylation states influence the fate of proteins. Phosphorylation of threonine by protein kinase Cβ prolongs the lifetime of FTO [129], while phosphorylation of specific serines of n‐terminal domains—S249 and S253—leads to degradation of FTO in mice [130], and phosphorylation of specific threonine T150 affects cellular localization of FTO and its ability to remove m6A from specific transcripts [83]. Phosphorylation may also indirectly affect the function of FTO, for example, by regulating its localization in cells, its interaction with other proteins, or its sensitivity to degradation through phosphorylation. Phosphorylation of mouse FTO results in ubiquitination [130]. It has been discovered that the PPI between FTO and CAM is crucial to the function of FTO in cell homeostasis. A reasonably conserved motif that is present in the FTO amino acid sequence and is anticipated to be recognized by CaM. The calcium‐binding region of CaM is made unstable by contact with FTO [131].

#### 3.5.2 Phosphorylation of ALKBH5

It was discovered that the complete stress response under hypoxic stress led to the production of ALKBH5, and that m6A methylation had a role in directing the substitution translation of mRNA [132]. ERK1/2 is activated by ROS, and this causes JNK to also become active. As a consequence, serine phosphorylation of ALKBH5—instead of tyrosine or threonine—was enhanced by active JNK. ALKBH5 that has been phosphorylated undergoes SUMO, which inhibits demethylase action and raises m6A levels all around. The process is that ALKBH5 phosphorylation increases ALKBH5's association with the SUMO E2 conjugase UBC9 and inhibits ALKBH5's interaction with SENP1, boosting ALKBH5's SUMO reaction in response to ROS stress [39].

### 3.6 Regulation of Phosphorylation on m6A Readers

#### 3.6.1 Phosphorylation of YTHDF2

The most frequent kind of adult malignant brain tumor is GBM, and YTHDF2 overexpression is clinically linked to a poor prognosis in glioma patients. The phosphorylation of serine 39 and threonine 381 in YTHDF2 by GFR/SRC/ERK signaling stabilizes the YTHDF2 protein. The growth, invasion, and tumorigenesis of GBM cells depend on YTHDF2. YTHDF2 decreases the lifespan of glioma patients by promoting the m6A‐dependent degradation of LXRA and HIVEP2mRNA. YTHDF2 mostly does this through downregulating LXR and HIVEP2 in GBM cells, which promotes tumorigenesis. Additionally, in GBM cells, YTHDF2 prevents LXR‐dependent cholesterol homeostasis [133].

#### 3.6.2 Phosphorylation of IGF2BP1

Signal transduction pathways control various biological processes, leading to different results. p38 MAPK is involved in regulating a wide range of cellular processes through phosphorylation activation of related signal molecules, and the disorder of p38 MAPK is related to various pathological conditions. Studies have shown that IGF2BP1 is an interacting protein of p38 MAPK, interacting with MAPK. The interacting proteins are phosphorylated by MAPK at the proline‐directed serine/threonine residue. Unlike other p38 MAPK substrates [134], IGF2BP1 is a cancerous fetal protein that is only expressed in cancer cells and will be a potential target for the development of new cancer drugs with fewer side effects [135].

As a result of phosphorylating AKT and regulating its activity, mTORC2 aids in cell survival [136]. The expression of c‐MYC is regulated at many levels, with posttranscriptional regulatory mechanisms controlling the majority of these levels. The fetal RNA binding protein IGF2BP1 regulates the posttranscriptional production of c‐MYC and prevents cleavage of the CRD region [137]. At Ser181, mTORC2 cotranslationally phosphorylates IGF2BP1. When IGF2BP1 is produced de novo during carcinogenesis and embryogenesis, mTORC2‐mediated phosphorylation of IGF2BP1 at Ser181 may serve as a feedback inhibitory mechanism that prevents IGF2BP1 from expressing itself [138]. To move the silent mRNA to the precise translation location and guarantee the proper production of the protein in space, Ser181 must be phosphorylated. On the other hand, IGF2 mRNA's IRES must first be phosphorylated in order for translation to begin.

#### 3.6.3 Phosphorylation of IGF2BP2

An important factor in the immortalization of tumor cells is mitochondrial oxidative phosphorylation. Instead of glycolysis, the majority of brain tumor cells produce energy by oxidative phosphorylation [139]. To change the balance of oxidative phosphorylation, get rid of damaged mitochondrial components, and control reactive oxygen species, mitochondrial dynamics is tightly synchronized with GBM biology [140]. According to studies, HOTAIRM1 controls GBM mitochondrial activity, including oxidative phosphorylation and serine metabolism. According to the molecular mechanism, HOTAIRM1 may improve PTBP1 and IGF2BP2's association, entice them to bind to SHMT2 mRNA, and then boost the quantity of SHMT2 protein by improving the stability of its mRNA, resulting in mitochondrial activity and the malignant growth of glioma [141].

### 3.7 Regulation of SUMOylation on m6A Pathway

One of several proteins that Ub is comparable to that has been found recently is called the SUMO. It establishes covalent connections with certain lysine branches of target proteins by a method akin to ubiquitination and SUMOylates them. It has been shown that SUMO may modify a variety of viral proteins, signaling molecules, and gene TFs. In contrast to ubiquitination, SUMOylation does not encourage the destruction of its target proteins. The role of SUMOylation in proteins involves modifying how proteins interact with one another and with cells, as well as increasing protein stability.

#### 3.7.1 SUMOylation of METTL3

SUMOs are joined to lysine residues unique to proteins via a process known as SUMOylation [142, 143]. It is crucial for a variety of cellular functions, including signal transmission, chromatin structure, gene expression, and genome maintenance. SUMOylation controls the location and function of proteins similarly to the nonproteolytic hydrolysis of Ub. Additionally, SUMO post translationally changed the nuclear targets of several signaling pathways, including TGF, WNT, and cytokines. It has been shown that SUMOylation sometimes faces competition from ubiquitination or acetylation for shared lysine residues.

According to studies, SUMO1 mostly modifies METTL3 on the lysine residues K177, K211, K212, and K215. The SUMO1‐specific protease SENP1 may decrease these SUMO residues. While METTL3's SUMOylation has no effect on its stability, location, or interaction with METTL14 and WTAP, it severely reduces the activity of the m6A methyltransferase and lowers the amounts of m6A in mRNAs [147]. The METTL14 MTDS and two zinc finger motifs (ZnF, 259–340) of the Cys–Cys–Cys (CCCH) type are crucial active sites for m6A methylation. The largest SUMO acceptor site, K177/211/212/215, is situated away from the MTDS but close to the two zinc F moths in the N‐terminal domain of METTL3. Its interaction with substrate mRNAs may be affected spatially by SUMO, which eventually reduces its m6A methyltransferase function.

#### 3.7.2 SUMOylation of IGF2BP2

**FIGURE 3:**
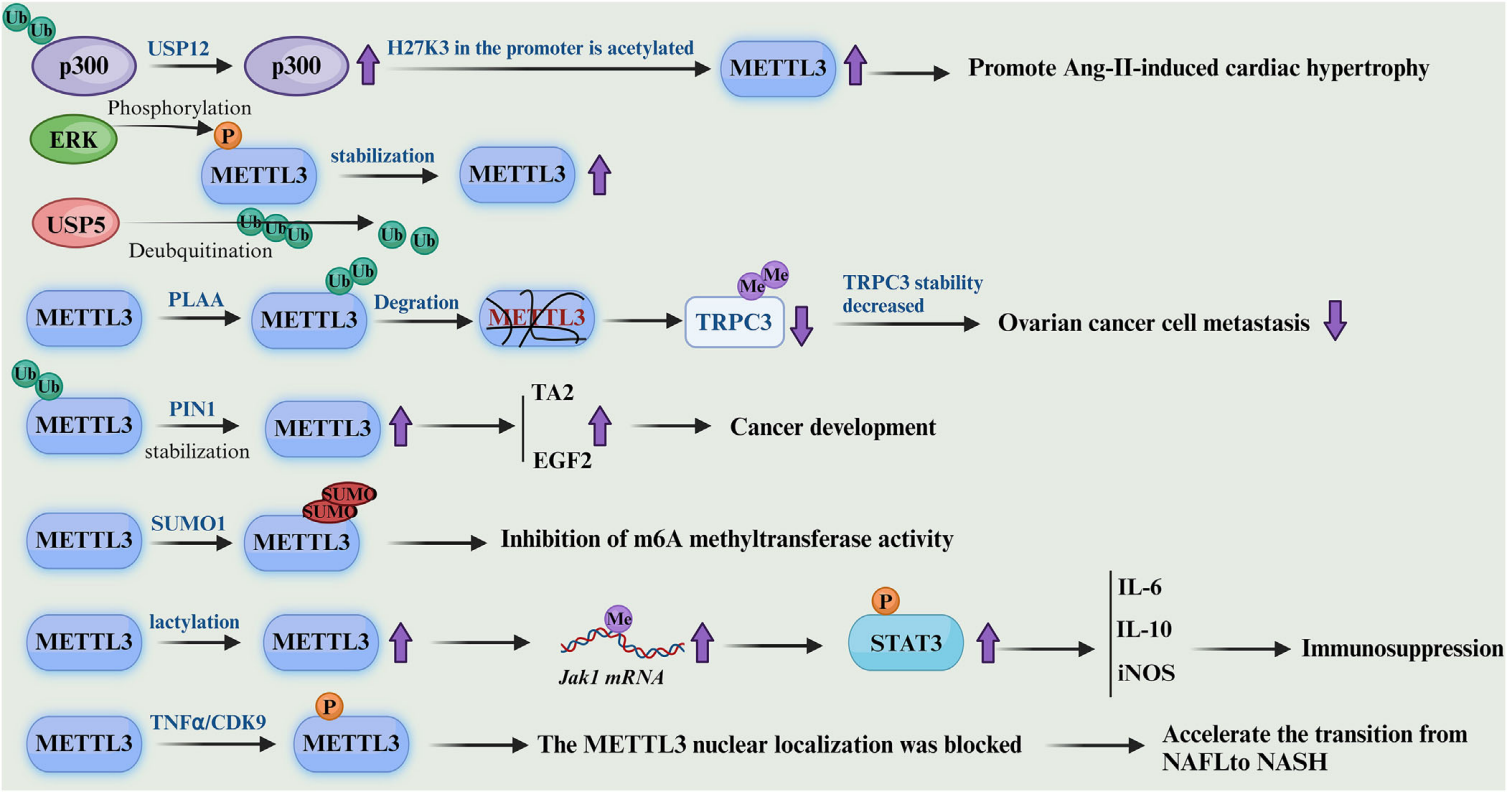
Posttranslational modifications associated with METTL3. *Stabilization*: ERK phosphorylation, PIN1 isomerization, USP5 deubiquitination. *Inhibition*: SUMOylation, lactylation (↓m6A activity, ↑immunosuppression). *Degradation*: PLA4 ubiquitination (↓TAB3). *Localization*: TNFα/CDK9 blocks nuclear entry. Outcomes span cardiac, cancer, immune and metabolic diseases.

The main mechanism of SUMO involves SUMO molecules binding to the lysine residues of the target protein's receptor. This is followed by three stages in an enzyme cascade that gradually modify the substrate: activation of the involved heterodimer E1 enzymes (SAE1 and SAE2/UBA2), coupling via the E2 enzyme UBC9, and substrate modification via E2 cooperation and E3 protein ligase. Substrate protein structure and function are regulated by this. IGF2BP2's main SUMO site is K497/505/509R, according to studies. This protein is mostly attached to SUMO1, which SENP1,a de‐SUMOylating enzyme (DeSumoEs) promoted. IGF2BP2's subcellular position is not much affected by SUMO, but it may promote the creation of IGF2BP2 protein, prevent its degradation, and ultimately improve its stability. SUMO increased IGF2BP2 expression and stability by blocking ubiquitination, and the OIP5–AS1/miR‐495‐3p axis further increased VM capacity in glioma cells. Treatment for gliomas requires further research, and the molecular targets miR‐495‐3p, OIP5–AS1, and IGF2BP2 could become apparent in the future [144] (Figure 3).

#### 3.7.3 SUMOylation of YTHDF2

One of the key readers of methylation, YTHDF2, is capable of identifying m6A‐modified RNA and causing its destruction. The C‐terminal YTH domain and an N‐terminal region rich in P/Q/N make up the structure of YTHDF2, which binds m6A and collects CNOT1 to degrade m6A‐modified RNA. According to certain research, the primary SUMO site of YTHDF2 is K571. Anoxic conditions cause YTHDF2 to become SUMOylated. Since SUMOylation modifies the activity, stability, localization, and protein‐protein interactions (PPIs) of its target proteins, it is widely accepted that this modification plays a role in a variety of cellular processes and signaling pathways. However, unlike many SUMOylated proteins, YTHDF2's SUMOylation has little impact on its localization or ubiquitination [145]. The binding affinity of YTHDF2 to the modified RNA's m6A region is increased by SUMOylation. Several malignancies, including those of the brain, lung, liver, pancreatic, lymphoma, and multiple myeloma, are accelerated [146, 147, 148, 149, 150, 151].

### 3.8 Regulation of O‐GlcNAc on m6A Pathway

One recently identified PTM is called O‐GlcNAc. Single O‐linked N‐acetylglucosamine (O‐GlcNAc) moieties bind to serine and threonine residues in cytoplasmic, nuclear, and mitochondrial proteins in this irregular glycation. O‐GlcNAc, n‐acetylglucosamine, uridine diphosphate (UDP‐GlcNAc), and glucose are the end products of nutrient fluxes via the hexosamine biosynthesis pathway, which integrates glucose, amino acids, fatty acids, and nucleotide metabolism. O‐GlcNAc signaling is not only reliant on the availability of nutrients, but it is also extremely vulnerable to several types of cellular stress, including as thermal shock, hypoxia, and nutritional restriction. The mechanisms governing this response are still being studied. O‐GlcNAc has therefore been suggested as a nutritional and stress sensor to control a variety of cellular functions, including signal transduction, metabolism, transcription, and translation [152, 153].

#### 3.8.1 O‐GlcNAc of YTHDF1, YTHDF2, YTHDF3

One of the most significant PTMs, O‐GlcNAc, mostly takes place over serine and threonine residues in proteins, is crucial for a number of cellular activities, including immune response, signal transmission, and cell metabolism. O‐GlcNAc is solely controlled by a few of enzymes that are hostile to one another, unlike other forms of protein glycosylation, and it does not affect more intricate glycan structures. The only enzymes that are now known to function as “writers” and “erasers” of mammalian O‐GlcNAc, respectively, are O‐GlcNAc transferase (OGT) and O‐GlcNAcase. Occupying the same amino acid residues, phosphorylation and O‐GlcNAc result in substantial crosstalk between the two PTMS with high dynamics. Its function has not received as much attention as other posttranslational alterations. A technique known as CHO–GlcNAc has been found recently. This technique was used to identify the probable O‐GlcNAc sites on two readers of m6A, YTHDF1 and YTHDF3 [154].

**FIGURE 4:**
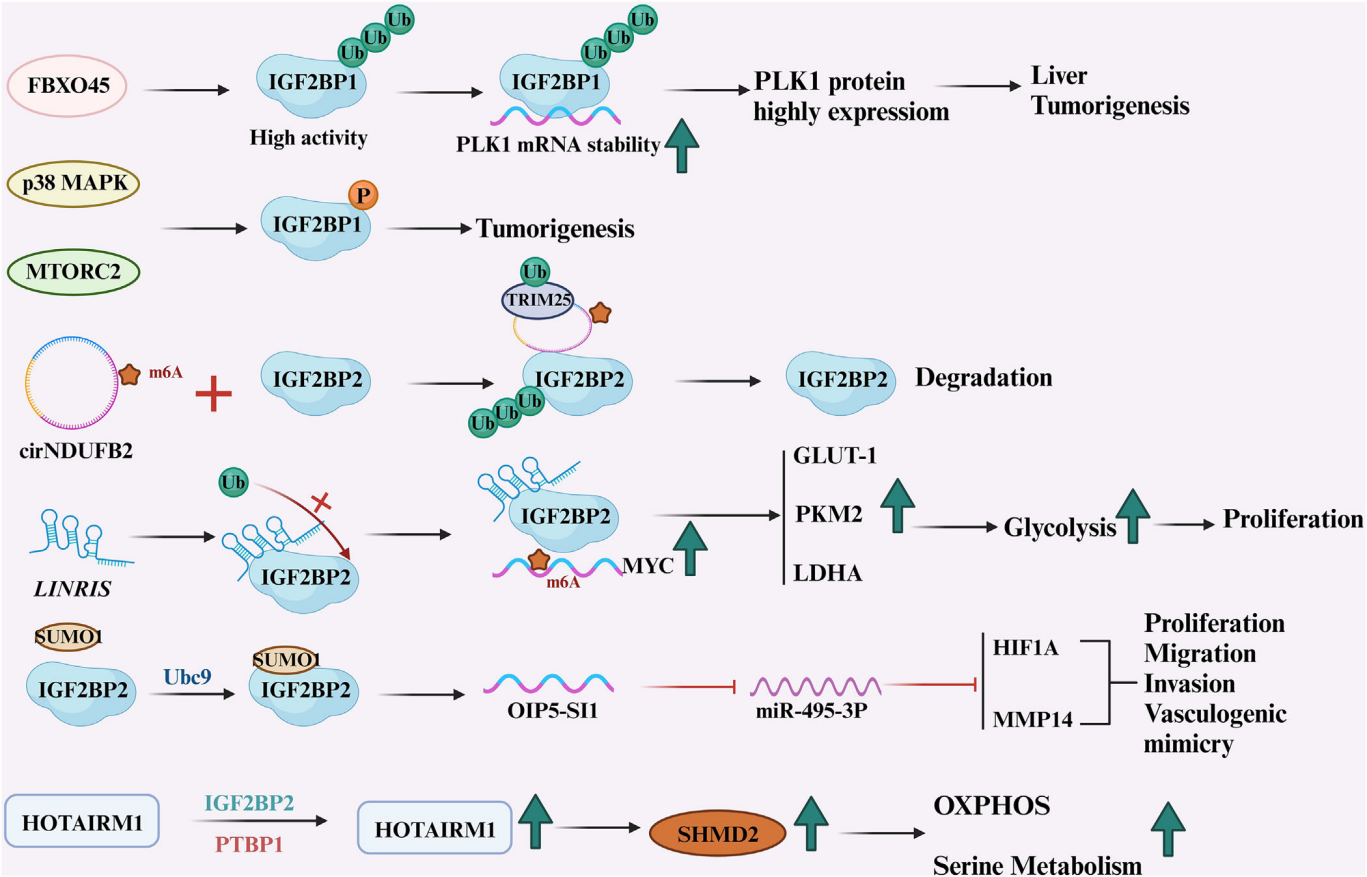
Posttranslational modifications associated with IGF2BP1 and IGF2BP2. This figure illustrates the posttranslational modifications (PTMs) occurring on IGF2BP1 and IGF2BP2 proteins, including phosphorylation, ubiquitination, and SUMOylation, which regulate target molecule activity.

O‐GlcNAc is a PTM that influences intracellular PPIs, stability, and activity. It connects nutrition fluxes to gene transcription in viral replication and cancer. After HBV infection, HBP fluxes and O‐GlcNAc levels greatly increased. YTHDF2 is one of the host proteins that may be O‐GlcNAcylated, and HBP overexpression following HBV infection enhanced YTHDF2 O‐GlcNAc. The mRNA stability of MCM2 and MCM5, which is reliant on m6A and may considerably suppress tumor growth and progression, is promoted by OGT‐mediated O‐GlcNAc of YTHDF2 at Ser263. Potential novel therapy for Hepatitis B virus‐related liver cancer may include focusing on OGT‐mediated YTHDF2 O‐GlcNAc [155] (Figure 4).

### 3.9 Regulation of Lactylation on m6A Pathway

Lactic acid is a compound produced during the Warburg effect and is widely regarded as an energy source and metabolic byproduct. Lactate‐derived histone lysine lactation is a novel epigenetic modification that directly stimulates gene transcription of chromatin. Recent studies have illuminated the critical roles of both lactylation and m6A modification in regulating tumor‐infiltrating myeloid cells (TIMs) and various tumor‐related processes. Lactate, a byproduct of glycolysis, accumulates in the tumor microenvironment and has been shown to induce epigenetic alterations that influence TIM function and promote immune evasion [156]. For instance, some research demonstrated that the upregulation of METTL3 in TIMs correlates with poor prognosis in colon cancer patients. METTL3 mediates m6A modification on Jak1 mRNA, enhancing the translation of JAK1 protein and subsequently activating the STAT3 signaling pathway. This process underscores the immunosuppressive capacity of TIMs enhanced by lactate‐driven METTL3 upregulation through H3K18 lactylation, which is critical for METTL3's RNA‐binding ability [157]. Similarly, lactate was found to facilitate p300‐mediated H3K18 lactylation at the METTL3 promoter, amplifying m6A modification levels and influencing the stability of target mRNAs such as ACSL4, through a YTHDC1‐dependent mechanism [158].

Moreover, the implications of lactylation extend beyond immune regulation; it has been implicated in promoting ferroptosis in alveolar epithelial cells during sepsis‐associated lung injury, highlighting a GPR81/H3K18la/METTL3/ACSL4 axis. In this context, the inhibition of METTL3 effectively alleviated hyperlactate‐induced ferroptosis, suggesting that targeting METTL3 could be a promising therapeutic strategy in inflammatory lung diseases [157]. Furthermore, in gastric cancer, elevated copper levels were associated with METTL16‐mediated cuproptosis, where lactylation of METTL16 was identified as a key regulatory mechanism that enhances its function in m6A modification of FDX1 mRNA. This finding suggests that the interplay between copper metabolism and lactylation could be leveraged for therapeutic strategies in copper‐rich tumors [159]. Interestingly, in the context of arsenite‐induced pulmonary fibrosis, lactylation and m6A modifications were found to regulate the secretion of TGF‐β1 through YTHDF1 and NREP, promoting fibroblast‐to‐myofibroblast transition. This emphasizes the role of lactylation in the crosstalk between epithelial cells and myofibroblasts, further demonstrating the relevance of lactylation in pathological conditions. Lastly, the role of histone lactylation in tumorigenesis has gained attention, particularly its association with poor prognosis in ocular melanoma. The lactylation‐mediated upregulation of YTHDF2 facilitates the degradation of m6A‐modified tumor suppressor mRNAs such as TP53, promoting tumor growth and progression. In summary, the integration of lactylation and m6A modifications presents a complex regulatory network that influences tumor immunity, progression, and responses to therapy. Understanding these interactions could unveil novel therapeutic targets and strategies for managing various cancers and related conditions.

#### 3.9.1 Lactylation of METTL3

**FIGURE 5:**
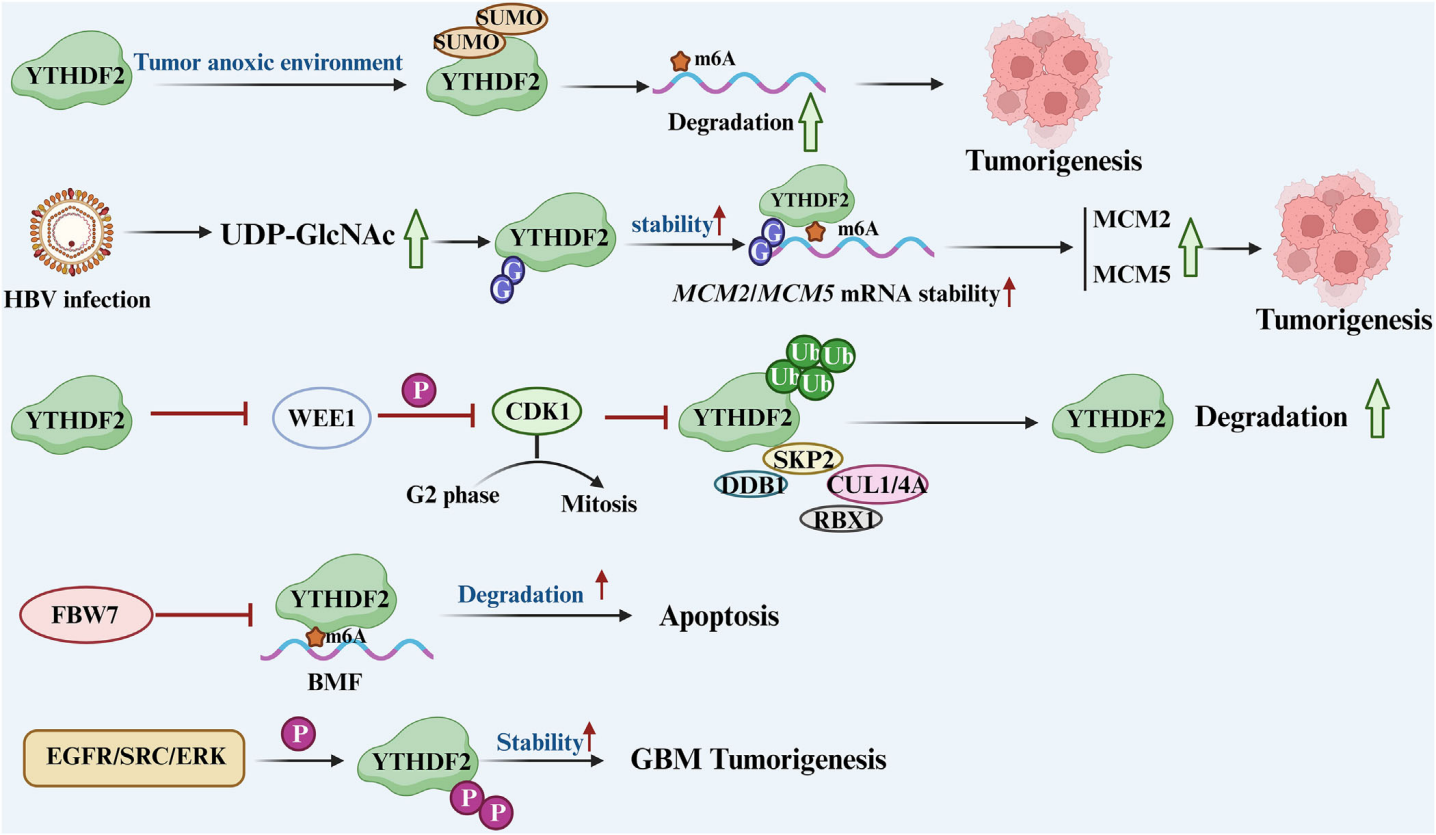
Posttranslational modifications associated with YTHDF2. This figure illustrates the posttranslational modifications (PTMs) occurring on YTHDF2 proteins, including phosphorylation, ubiquitination, and SUMOylation, which regulate target molecule activity.

Tumor infiltrating bone marrow cells (TIM) are important cell groups involved in tumor immune escape. Lactate accumulated in the tumor microenvironment upregulates the expression of METTL3 in TIM by lactating H3K18, thus methylating Jak1 mRNA, enhancing the translation efficiency of Jak1 protein and increasing its phosphorylation level. Promote tumor development through the Jak1–STAT3 axis [158] (Figure 5).

#### 3.9.2 Lactylation of FTO

Diabetic retinopathy (DR) is a major cause of irreversible vision loss in working‐age individuals. FTO, an m6A demethylase, plays a role in energy regulation, but its impact on DR is not well explored. This study found increased FTO expression in the vitreous fibrovascular membranes of patients with proliferative DR. FTO promoted cell cycle progression and tip cell formation in ECs, enhancing angiogenesis in vitro, in mice, and in zebrafish. Additionally, FTO regulated interactions between ECs and pericytes, leading to diabetic microvascular leakage, and also mediated EC‐microglia interactions, contributing to retinal inflammation and neurodegeneration. Mechanistically, FTO influenced EC functions by stabilizing CDK2 mRNA in an m6A‐YTHDF2‐dependent manner. Lactate‐induced histone lactylation was identified as the driver of FTO upregulation under diabetic conditions. The FTO inhibitor, FB23‐2, reduced angiogenic phenotypes in vitro, and a nanoparticle delivery system for FB23‐2 demonstrated therapeutic potential in mice. Overall, this study highlights FTO's crucial role in DR and suggests it as a potential therapeutic target [160].

### 3.10 Regulation of Arginine Methylation on m6A Pathway

S‐adenosylmethionine (SAM) is a donor methyltransferase that is used in biomethylation to add methyl groups to proteins, RNA, and DNA, among other metabolites and biopolymers. The majority of protein methylation takes place at the polypeptide's amino terminal alpha‐amino group or at the nitrogen atom of the basic arginine or lysine side chain. The most basic amino acid, arginine, can be modified posttranslationally by methylating it. This results in a change to the guanidine group, which is protonated at physiological pH. Numerous significant intramolecular and intermolecular interactions, including as hydrogen bonding and π stacking, are mediated by positively charged guanidine side chains.

#### 3.10.1 Arginine Methylation of METTL14

METTL14 has undergone another posttranslational change. Methylation of METTL14's arginine 255 (R255) facilitates the protein's binding to its RNA substrate. Nine protein arginine methyltransferases (PRMTs) catalyze the important posttranslational alteration known as protein arginine methylation. Asymmetric dimethylation and monomethylation are catalyzed by type I PRMTS, symmetrical dimethylation and monomethylation are catalyzed by type II PRMTS, and monomethylation is the sole reaction that type III PRMTS catalyzes. 85% of the activity of arginine methyltransferase is attributed to PRMT1. By controlling how proteins attach to other proteins and nucleic acids, particularly RNA, arginine methylation of proteins may control their function. Protein arginine N‐methyltransferase 1 (PRMT1) is the enzyme that mediates the methylation process. The m6A MTC's binding to its substrate RNA is stabilized by the arginine methylation of METTL14; when PRMT1 expression declines, the binding of METTL14 to WETP becomes weaker. Smad6/7 and Klf4 are downstream targets of R255 activity that are less methylated and more stable in R255K cells. The differentiation of R255K cells is influenced by the lifetime extension of these genes and their regulatory networks. These findings imply that METTL14 R255 methylation is necessary for proper cell fate transition in MESCs, particularly during endoderm development [119].

### 3.11 Cancers Associated and Therapy Involvement with PTM of m6A

#### 3.11.1 Ubiquitination

PIN1 and METTL3 interact to stop METTL3 from being degraded by Ub‐dependent proteasomes and lysosomes. Because it stabilizes METTL3, more m6A modification of transcription coactivators with PDZ‐binding motifs (TAZ) and EGFR mRNA is increased, which improves translation. For breast cancer, the PIN1/METTL3 axis may offer an alternative therapeutic target [161]. Patients with severe ESCC frequently have radiotherapy failure, which is linked to CSC persistence. TRIB2 attaches itself to METTL14 and encourages the proteasomes mediated by COP1 to degrade METTL14. Positive feedback in ESCC is generated via the METTL14/miR‐99a‐5p/TRIB2 regulation system. Cancer dryness and radiological resistance in ESCCs are encouraged by this feedback circuit epigenetic suppression of p2 via Akt/mTOR/S21K6/HDAC1. For ESCC patients, these groups offer viable and efficient targets for anticancer treatment [49]. By deubiquitinating ALKBH5, USP36 stabilized it and caused the ALKBH36 target gene to become more highly expressed. Additionally, the USP5–ALKBH5 axis fragmentation reduced the GSCS's susceptibility to drugs as well as its ability to proliferate and renew itself. We propose that USP3A‐induced activation of ALKBH5 may serve as a foundation for the development of more potent glioma treatments, given the important functional significance of ALKBH5 in GBM carcinogenesis [162]. FBW7 increases YTHDF2's ubiquitination and proteolytic degradation. Ovarian cancer and the FBW7–YTHDF2–BMF axis. Carcinogenic YTHDF2 is degraded by FBW7 via interaction, which stabilizes m6A‐modified mRNA, including the pro‐apoptotic gene BMF, and impairs the survival and proliferation of ovarian cancer cells. The findings also highlight the axis's therapeutic importance, which will guide the creation of new anti‐cancer treatments in the future [92]. By preventing EFTUD2 from ubiquitinating the YTHDF3 protein, PFKL positively controls the expression of YTHDF3. EFTUD2 may function as an enzyme that modifies Ub during the degradation of the YTHDF3 protein and as a scaffold in interactions between proteins. The establishment and progression of HCC are facilitated by YTHDF3, and inferior overall survival in HCC patients is predicted by YTHDF3 overexpression [96]. Beyond only creating TRIM2, circNDUFB2 IGF2BPs Ub degradation is aided by circNDUFB25/IGF2BPs ternary complexes, but they also stimulate cellular immunity by activating RIG‐I. By causing disruptions to stable IGF2BPs and triggering the immune system, circNDUFB2 prevents the advancement of NSCLC. When added to vaccines, exogenous circRNAs can boost their effectiveness and stimulate T cells that are specific to a certain antigen. With its powerful RIG‐I agonist properties, circNDUFB2 may hold great promise for immunotherapy in non‐small cell lung cancer [99].

#### 3.11.2 Phosphorylation

The process of DSBR is intricate and involves several cellular elements. ATM phosphorylates METTL3, which then becomes active in reaction to DSB. The m6A modification of RNA at DSB is catalyzed by activated METTL3, and the buildup of DNA‐RNA hybrids on DSB is increased by the m6A reading protein YTHDC1, which also shields the m6A‐modified RNA. An essential pathway to raise DSBR efficiency in normal cells is METTL3–m6A–YTHDC1. On the other hand, blocking this pathway could make radiation and chemotherapy‐based cancer treatments more successful [163]. METTL3 may contribute to the development of resistance to chemotherapy and/or radiation‐based cancer treatments [124]. a particular subset of neutrophils called C5aR1 neutrophils, which are linked to poor survival and tumor development in BC patients. The synergistic activation of ERK1/2 signaling by TNF‐α and IL1β released by C5AR1‐positive neutrophils phosphorylates WTAP at serine 341 to stabilize the WTAP protein. WTAP stability also influenced BC cells' glycolysis activity and encouraged the methylation of RNA m6A in ENO1. These results demonstrate the WTAP–ENO1 network and C5aR1 neutrophils’ potential as BC treatment targets [127]. Because GBM cells rely heavily on cholesterol for life, they perish when LXR is activated. The level of YTHDF2 and the level of LXRA in the TCGA glioma data set are negatively linked. The phosphorylation and stabilization of YTHDF2 protein by EGFR/SRC/ERK inhibits the expression of the YTHDF2 target gene. These results provide insight into the process behind YTHDF2 overexpression in GBM as well as the function of EGFR signaling in carcinogenesis. LXRA and HIVEP2 mRNA clearance, mediated by YTHDF2 and reliant on m6A, is necessary for cholesterol dysregulation, cell division, invasion, and GBM carcinogenesis [133].

#### 3.11.3 O‐GlcNAcylation

The O‐GlcNA acylation of YTHDF2 did not change its intracellular localization or binding affinity, but enhanced the stability of YTHDF2 protein. The S263A mutation leads to the loss of O‐GlcNAcylation and promotes degradation by shortening the half‐life of YTHDF2 by increasing its K48‐linked ubiquitination. The S263A mutant largely suppresses the carcinogenic effect of YTHDF2 in HBV‐associated HCC. Inhibition of HCC progression by targeting YTHDF2 or YTHDF2 O‐GlcNAc may be a novel therapeutic strategy for HBV‐related HCC intervention [155].

#### 3.11.4 SUMOylation

Through regulating the OIP5–AS1/miR‐495‐3p axis, SUMOylation of IGF2BP2 increases angiogenesis simulation of glioma. In glioma tissues and cells, there was an upregulation of IGF2BP2 and OIP5–AS1, and a downregulation of miR‐495‐3p. The primary mechanism via which IGF2BP2 SUMOylation is triggered is interaction with SUMO1. SUMOylation of IGF2BP2 is a crucial process in the control of VM in gliomas. OIP5–AS1, miR‐495‐3p, and IGF2BP2 could represent potential targets for glioma molecular targeted treatment [144] (Table 1).

**TABLE 1:** Diseases and physiological processes associated with posttranslational modification of m6A methylated elements.

Posttranslational modification	Modified molecule	Related disease or related physiological processes	References
Ubiquitination	METTL3	Pathological cardiac hypertrophy, ovarian cancer, breast cancer	[164], [161], [56]
	METTL14	Liver injury, esophageal cancer, bladder cancer	[48], [49], [51]
	WTAP	Diet‐induced obesity, viral infection	[62], [54]
	ALKBH5	Glioblastoma	[88]
	FTO	Breast cancer, cervical cancer, melanoma, ovarian cancer, hepatocellular carcinoma, renal malignancy, colorectal cancer, ischemic stroke	[68], [69], [70], [71], [72], [74]
	YTHDF1	Sepsis	[91]
	YTHDF2	Ovarian cancer, AML	[92], [95]
	YTHDF3	HCC	[96]
	IGF2BP1	HCC	[97]
	IGF2BP2	Colorectal cancer	[99], [106]
	IGF2BP3	Gliomas, central nervous system malignancies	[107], [109]
Phosphorylation	METTL3	NASH, DSBS	[124], [126]
	METTL14	Alpha herpesvirus infection	[117]
	WTAP	Breast cancer	[127]
	FTO	Human neuroblastoma	[165]
	ALKBH5	Reactive oxygen species (ROS)‐induced oxidative stress	[39]
	YTHDF2	Glioblastoma	[133]
	IGF2BP1	Various cancers	[135]
	IGF2BP2	Glioma	[141]
SUMOylation	METTL3	Human non‐small cell lung carcinoma	[143]
	YTHDF2	Brain, lung, liver, pancreatic, lymphoma, and multiple myeloma malignancies	[146, 147, 148, 149, 150, 151]
	IGF2BP2	Glioma	[144]
Lactylation modification	METTL3	Tumor immune escape	[158]
Arginine methylation	METTL14	mESC differentiation	[166]
O‐GlcNAcylation	YTHDF1	Substantial crosstalk between phosphorylation and O‐GlcNAcylation with high dynamics	[154]
	YTHDF2	Hepatitis B virus‐related liver cancer	[155]
	YTHDF3	Substantial crosstalk between phosphorylation and O‐GlcNAcylation with high dynamics	[154]

## 4 RNA Modifications in Diseases

### 4.1 Cancer

#### 4.1.1 m6A and Tumorigenesis

**FIGURE 6:**
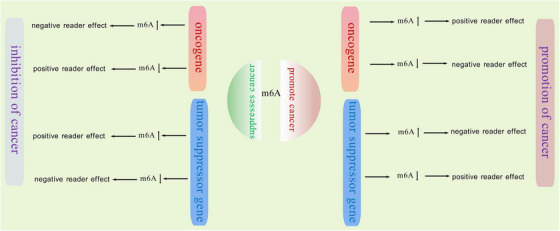
Dual roles of m6A readers in cancer inhibition and promotion via oncogene regulation. Blue: Promote oncogenes in tumor subtypes. Red: Inhibit tumor suppressors. Dashed arrows show cancer‐relevant pathways. Outcomes are microenvironment dependent.

The role of m6A in cancer has been extensively investigated, revealing its dual and context‐dependent functions. In numerous cancer types, m6A modifications are dysregulated, with alterations in the expression and activity of “writer, ” “eraser, ” and “reader” proteins. For instance, in AML, the m6A demethylase FTO is overexpressed, leading to decreased m6A levels on specific transcripts [167]. This, in turn, stabilizes oncogenic transcripts such as MYC and BCL2, promoting cell proliferation and survival. Conversely, in breast cancer, increased METTL3‐mediated m6A methylation has been associated with enhanced tumorigenicity, potentially through modulating the stability and translation of genes involved in cell cycle progression and metastasis [168]. Moreover, m6A modifications can influence the tumor microenvironment by affecting immune cell function and cytokine production. The complex interplay between m6A and cancer progression positions it as a promising therapeutic target, with ongoing efforts to develop small molecule inhibitors or modulators of m6A‐regulating enzymes. In seminoma, the mRNA levels of VIRMA and YTHDF3 are both high and positively correlated. Considering their roles as m6A writers and readers respectively, it is easy to speculate whether m6A may contribute to the emergence and maintenance of the SE phenotype [169]. Accumulating evidence shows that m6A plays a dual role in cancer [170]. On the one hand, m6A regulates the expression of oncogenes or tumor suppressor genes, thereby affecting the progression of tumors. On the other hand, it can regulate the level of m6A and the expression and activity of m6A enzymes, thereby influencing the role of m6A in cancer. In lung adenocarcinoma, the expression level of METTL3 is significantly upregulated. METTL3 enhances the translation of the oncogene BRD4 by forming an mRNA loop with EIF3, thereby promoting ribosome recycling and facilitating the translation process of the oncogene. These findings indicate that METTL3 not only functions as an m6A methyltransferase but also participates in postmethylation regulation of target mRNA, acting as a reader in this context [171]. In liver cancer, the levels of METTL3 and YTHDF1 are significantly elevated, which is associated with poorer overall survival. The m6A modification in the coding sequence of Snail enhances Snail expression via YTHDF1‐mediated translation. Snail is a key TF involved in the epithelial–mesenchymal transition (EMT). A reduction in m6A levels, caused by the loss of METTL3, impairs the EMT process in cancer cells [172]. In breast cancer, the expression of METTL3 is significantly upregulated. HBXIP (hepatitis B X‐interacting protein) plays a critical role in promoting the malignant phenotypes of breast tumors. METTL3 positively regulates HBXIP expression. Notably, HBXIP also enhances METTL3 expression by inhibiting miRNA let‐7 g, which negatively regulates METTL3 expression through targeting its 3′ UTR [173] (Figure 6).

#### 4.1.2 Other Modifications in Cancer

Beyond m6A, other RNA modifications also contribute to cancer development. m5C modifications, mediated by enzymes like NSUN2 and DNMT2, have been implicated in cancer cell metabolism and metastasis. Studies have shown that in non‐small cell lung cancer, RNA m5C hypermethylation and NSUN2 are significantly associated with intrinsic resistance to EGFR‐TKIs. Overexpression of NSUN2 leads to gefitinib resistance and tumor recurrence, while genetic inhibition of NSUN2 causes tumor regression and overcomes the intrinsic resistance to gefitinib both in vitro and in vivo [174]. In a study, researchers found that RNA methyltransferase NSUN2 was highly expressed in GC and served as a prognostic biomarker associated with poor prognosis. Downregulation of NSUN2 can inhibit the proliferation and metastasis of GC cells in vitro, while overexpression of NSUN2 can promote the proliferation and metastasis of GC cells [175]. In colorectal cancer, aberrant m5C patterns have been observed, potentially affecting the expression of genes involved in EMT and tumor invasion. m1A modifications, which are prominent in mitochondrial tRNAs, can impact mitochondrial translation and energy metabolism, providing a selective advantage to cancer cells. Additionally, pseudouridylation and 2′‐O‐methylation have been shown to play roles in cancer progression, albeit with less well‐understood mechanisms. The identification of specific enzymes responsible for these modifications has spurred research into developing targeted therapies, as modulating these enzymes could potentially disrupt cancer cell growth and survival pathways [176].

### 4.2 Neuropsychiatric Disorders

#### 4.2.1 m6A and Related Modifications in Mental Illness

RNA modifications, particularly m6A, have emerged as critical regulators in neuropsychiatric disorders. In depression, alterations in m6A levels and the expression of associated enzymes have been reported. For example, decreased FTO expression in the hippocampus of depressed patients and animal models has been linked to increased m6A methylation on specific mRNAs, affecting synaptic plasticity and neurotransmitter signaling. In autism spectrum disorders (ASD), aberrant m6A patterns have been observed in genes related to neuronal development and synaptic function, suggesting a role in the disrupted neurodevelopmental pathways characteristic of ASD [177]. In Alzheimer's disease, m6A modifications have been implicated in the regulation of amyloid precursor protein metabolism and tau phosphorylation, key pathological processes in the disease. These findings highlight the potential of RNA modifications as biomarkers and therapeutic targets for these debilitating disorders [178].

#### 4.2.2 Implications for Therapeutics

Targeting RNA modifications holds promise for the development of novel therapeutics for neuropsychiatric disorders. Modulating the activity of m6A “writers, ” “erasers, ” or “readers” could potentially correct aberrant gene expression patterns underlying these diseases. Diclofenac (MA) is a selective FTO inhibitor by competing with the FTO binding site. As an ethyl ester derivative of MA, MA2 inhibits the growth and self‐renewal of GSCS in vitro and tumor growth in vivo [179, 180]. As another inhibitor of FTO, FB23‐2 impairs the proliferation of AML cells and enhances the differentiation of AML cells [181]. In epithelial ovarian cancer, elevated m6A levels upregulate the Wnt signaling pathway by enhancing the stability of FZD10, leading to resistance to poly (ADP‐ribose) polymerase inhibitors (PARPi). Therefore, the combination of inhibiting the Wnt signaling pathway and PARPi may be a potential therapeutic strategy for EOC [182]. In GBM, upregulated METTL3 promotes the maintenance of GSCs and the progression of GBM. The deletion of METTL3 leads to an increased sensitivity of GSCS to γ irradiation [183].

### 4.3 Cardiovascular Diseases

CVD is the leading cause of death worldwide. Recent studies have revealed the significant role of RNA modification in the progression of heart failure, which provides a new approach for developing intervention measures and strategies for the treatment of heart failure.

#### 4.3.1 m6A and Cardiovascular Health

In the cardiovascular system, m6A modifications play a crucial role in maintaining heart function and vascular homeostasis. m6A has been shown to regulate the expression of key proteins involved in cardiac contractility, such as sarcomeric proteins and ion channels. Dysregulation of m6A in cardiomyocytes can lead to abnormal cardiac hypertrophy and heart failure. In vascular smooth muscle cells, m6A modifications influence cell proliferation, migration, and phenotype switching, processes that are central to the development of atherosclerosis and restenosis [184]. For example, alterations in m6A levels on transcripts encoding growth factors and cytokines can impact vascular remodeling. Understanding the role of m6A in CVDs offers potential therapeutic strategies, such as targeting m6A‐regulating enzymes to modulate gene expression and prevent or treat heart and vascular disorders [185]. Two‐thirds of the SMAD family members interact with METTL3, METTL14, and WTAP in human pluripotent stem cells to control pluripotency and differentiation. The activated classic TGF‐β and SMAD2/3 pathways have been proven to enhance cardiac fibrosis by promoting the differentiation of fibroblasts into myofibroblasts [38]. Interestingly, METTL14/ALKBH5 forms a positive feedback loop with the RNA‐binding protein HuR to regulate the stability of transcripts involved in cell cycle regulation and angiogenesis. Previously, we have noticed that myocardial infarction leads to the overexpression of HuR protein. HuR protein plays an important role in the stability of many inflammatory genes, namely, TNFα and TGF‐β. However, the roles of m6A methylation and the METTL14/ALKBH5–HuR axis in CVDs have not yet been explored [186]. Compared with m6A methylase, the role of demethylase in the cardiovascular system has been insufficiently studied. It is reported that the expressions of FTO and ALKBH5 are decreased in failing human hearts [187]. Studies have shown that in failing hearts, m6A hypermethylation regulates the translation of certain mRNA transcripts, which are mainly involved in metabolism and other important regulatory pathways [188].

#### 4.3.2 Emerging Research on Other Modifications

Recent studies have begun to explore the roles of other RNA modifications in CVDs. m5C modifications in mitochondrial tRNAs have been implicated in regulating mitochondrial function and energy production in the heart. In ischemic heart disease, changes in m5C patterns may contribute to mitochondrial dysfunction and cardiomyocyte death. Studies have found that in ApoE‐deficient mice, the deletion of the ICAM‐1 gene reduces monocyte recruitment, thereby preventing atherosclerotic lesions, and tRNA methyltransferase NSUN2 responds to Myc activation to promote cell proliferation. Recent studies have shown that homocysteine‐induced NSUN2 methylates ICAM‐1 mRNA and promotes ICAM‐1 translation. NSUN2 plays a role in vascular inflammation and ischemic necrosis of allograft by increasing the level of ICAM‐1 [175]. Acetylation of cytosine at the N4 position (ac4C) in mRNA has also emerged as a potential regulator in the cardiovascular system. Ac4C modifications have been shown to affect mRNA stability and translation efficiency, with implications for cardiac fibrosis and hypertrophy. Additionally, pseudouridylation in ribosomal RNAs has been associated with altered translational fidelity in cardiomyocytes, potentially influencing protein synthesis and heart function. These emerging findings suggest that a comprehensive understanding of multiple RNA modifications is essential for deciphering the complex pathophysiology of CVDs and developing targeted therapeutic interventions [189].

## 5 RNA Modifications in Health

### 5.1 Developmental Biology

#### 5.1.1 Role of RNA Modifications in Embryonic Development

RNA modifications play a pivotal role in embryonic development, orchestrating the precise and complex processes that transform a single fertilized egg into a fully formed organism. During embryogenesis, pluripotent stem cells undergo a series of tightly regulated differentiation events to give rise to diverse cell lineages, which ultimately form various tissues and organs. RNA modifications, such as m6A, m5C, and pseudouridylation, act as crucial regulators in this process. Thanks to the development of sequencing technology, m6A has been discovered to regulate the tumorigenesis process, including tumor proliferation, invasion and metastasis, by regulating oncogenes and tumor suppressor genes, and to affect oocyte maturation and embryonic development by regulating maternal and zygotic genes. The related proteins of m5C have been identified to be involved in the M5C‐dependent mode of embryonic development, plant growth and neural stem cell differentiation. m1A has also been revealed to be involved in these developmental processes. m7G dysregulation mainly involves neurodevelopmental disorders and neurodegenerative diseases [1, 190]. During early embryonic development, the composition and utilization of the cellular transcriptome must respond to temporal cues. Master TFs activate hundreds of transcripts, thereby shaping the cellular mRNA pool. During the selected TF transcriptional activation process, RNA modifications can be selectively deposited onto a set of transcripts. This modification provides an additional “identity” for the coordinated translation and decay of these transcripts, thereby facilitating the coordinated utilization and transition of the transcriptome. Therefore, it can be inferred that mRNA m6A methylation provides a mechanism that promotes rapid transcriptome renewal during the process of cell differentiation. During the transition from the mother to the zygote in zebrafish, the mother transcript rapidly degrades and the transcriptome is replaced by newly synthesized zygote mRNA. A portion of the maternal mRNA was methylated by m6A and rapidly cleared by YTHDF2. In the absence of YTHDF2, this clearance is delayed, preventing the timely initiation of the transformation and thereby leading to prolonged developmental delay [191, 192].

m6A modifications have been shown to be highly dynamic during embryonic development. For instance, in mouse embryos, the levels of m6A‐modifying enzymes, including METTL3 and FTO, change significantly at different developmental stages. METTL3‐mediated m6A methylation is essential for the proper differentiation of embryonic stem cells (ESCs) [193]. Depletion of METTL3 in ESCs leads to impaired differentiation potential, as it affects the stability and translation of key transcripts involved in lineage specification. In zebrafish embryos, m6A modifications have been implicated in the regulation of germ layer formation. Knockdown of METTL3 results in abnormal development of the mesoderm and endoderm, highlighting its critical role in early embryogenesis [194].

m5C modifications also contribute to embryonic development. In mammalian embryos, m5C is enriched in maternal mRNAs and plays a role in the maternal‐to‐zygotic transition (MZT). During MZT, the maternal mRNA pool is selectively degraded and replaced by zygotic transcripts [174]. m5C modifications on maternal mRNAs can prevent their premature decay, ensuring the timely and proper transition to zygotic gene expression. In addition, m5C modifications in mitochondrial tRNAs have been shown to influence mitochondrial function, which is crucial for providing energy during embryonic development.

Pseudouridylation, another prevalent RNA modification, is involved in ribosome biogenesis and function. In embryos, proper ribosome assembly and activity are essential for protein synthesis, which drives cell growth and differentiation. Ψ synthase enzymes catalyze the conversion of uridine to Ψ in rRNAs, enhancing ribosome stability and translational fidelity. Mutations in genes encoding Ψ synthases have been associated with developmental defects in model organisms, emphasizing the importance of this modification in embryogenesis [195].

These RNA modifications do not act in isolation but interact with other epigenetic mechanisms, such as DNA methylation and histone modifications, to coordinate gene expression programs during embryonic development. Dysregulation of RNA modifications during this critical period can lead to developmental disorders and congenital anomalies. Understanding the precise roles and mechanisms of RNA modifications in embryogenesis provides valuable insights into the fundamental processes of life and may offer potential therapeutic targets for treating developmental diseases.

#### 5.1.2 Impact on Adult Tissue Homeostasis

In adult organisms, RNA modifications continue to play a crucial role in maintaining tissue homeostasis. Tissue homeostasis requires a delicate balance between cell proliferation, differentiation, and apoptosis to ensure proper tissue function and repair. RNA modifications contribute to this balance by regulating gene expression in adult stem cells and differentiated cells.

Muscle stem cells are necessary for the homeostasis and regeneration of mammalian skeletal muscle. NA m6A modification plays a key role in muscle development and regeneration. Studies have found that m6A reader YTHDC1 is essential for the regeneration of mouse skeletal muscle and the proliferation of muscle stem cells. In the absence of YTHDC1, the muscle stem cells of adult mice cannot withdraw from the quiescent state. Mechanism speaking, YTHDC1 binds to M6A‐modified Pi4k2a and Pi4kb mRNA to regulate their selective splicing, thereby regulating PI4K‐Akt‐mTOR signaling. Ythdc1‐deficient muscle stem cells show deficiencies of phosphatidylinositol 3, 4, 5‐triphosphate, phosphorylated Akt and phosphorylated S6, which are associated with failed exit from quiescence [196]. It is known that the clearance of damaged mitochondria through mitochondrial autophagy is crucial for cellular homeostasis. In the heart, an organ with highly active metabolism, studies have found that the depletion of RBX2 inhibits mitochondrial ubiquitination and turnover, damages mitochondrial membrane potential and respiration, increases cardiomyocyte death, and has an overall impact on the mitochondrial proteome. In vivo, the deletion of the Rbx2 gene in the hearts of adult mice inhibits mitochondrial autophagy activity, triggers the accumulation of damaged mitochondria in the myocardium, and disrupts myocardial metabolism, leading to the rapid development of dilated cardiomyopathy and heart failure. Similarly, ablating RBX2 in a developing heart can lead to dilated cardiomyopathy and heart failure. The role of RBX2 in mitochondria is not dependent on Parkin. The deletion of the Parkin gene has no effect on the occurrence and progression of cardiomyopathy in RBX2‐deficient hearts. Furthermore, RBX2 controls the stability of PINK1 (PTEN‐induced kinase 1) in mitochondria [197]. The absence of YTHDF1 in adult cardiomyocytes leads to hypertrophy, fibrosis and dysfunction [198].

### 5.2 Immune Response

#### 5.2.1 RNA Modifications in Innate Immunity

The innate immune system serves as the first line of defense against invading pathogens, and RNA modifications play a critical role in modulating innate immune responses. Innate immune cells, such as macrophages, dendritic cells, and natural killer cells, express pattern recognition receptors (PRRs) that detect pathogen‐associated molecular patterns and initiate immune responses. RNA modifications can influence the function of these cells and the overall innate immune response in multiple ways [199].

m6A modifications have been shown to regulate the expression and function of PRRs. For example, in macrophages, m6A modification of the mRNA encoding Toll‐like receptor 3 affects its stability and translation, thereby modulating the cell's ability to respond to viral double‐stranded RNA [200]. Additionally, m6A modifications in the transcripts of interferon‐stimulated genes can regulate their expression, influencing the antiviral state of the cell. In dendritic cells, m6A modifications have been implicated in antigen presentation. They can regulate the processing and presentation of antigens, thereby affecting T cell activation and the initiation of adaptive immune responses [201].

Other RNA modifications also contribute to innate immunity. Pseudouridylation in rRNAs has been shown to enhance ribosome function in immune cells, leading to increased protein synthesis during an immune response [202]. In macrophages, 2′‐O‐methylation of RNA can affect the recognition of self and non‐self RNA. Alterations in 2′‐O‐methylation patterns can trigger innate immune activation, as the modified RNA may be recognized as foreign by PRRs. m5C modifications in tRNAs and mRNAs have been associated with the regulation of cytokine production in immune cells, which is crucial for coordinating the inflammatory response [203].

RNA modifications also play a role in the regulation of inflammasomes, which are multiprotein complexes that mediate the production of pro‐inflammatory cytokines. For instance, m6A modifications in the transcripts of genes involved in inflammasome assembly and activation can influence the magnitude and duration of the inflammatory response. Dysregulation of RNA modifications in innate immunity has been implicated in autoimmune diseases and chronic inflammatory disorders, highlighting their importance in maintaining immune homeostasis.

#### 5.2.2 Adaptive Immunity and RNA Modifications

The adaptive immune system provides a highly specific and long‐lasting defense against pathogens. RNA modifications are involved in various aspects of adaptive immunity, including the development, activation, and function of T and B lymphocytes.

In T cell development, RNA modifications play a crucial role in thymic selection and the differentiation of T cell subsets. m6A modifications have been shown to regulate the expression of key TFs involved in T cell fate determination. For example, in the differentiation of CD4+ T helper cells, m6A modifications can influence the expression of cytokines and cytokine receptors, determining the functional phenotype of the T cells. In addition, m6A modifications in the transcripts of T cell receptor (TCR) genes can affect TCR repertoire diversity, which is essential for recognizing a wide range of antigens [204].

During T cell activation, RNA modifications rapidly modulate gene expression to support the effector functions of T cells. m6A modifications can enhance the translation of mRNAs encoding cytokines, such as interleukin‐2, which is crucial for T cell proliferation and activation. Moreover, RNA modifications in noncoding RNAs, such as microRNAs and long noncoding RNAs, can regulate T cell activation and differentiation by modulating the expression of target genes [205].

In B cells, RNA modifications are involved in antibody production and class switching. m6A modifications have been shown to regulate the expression of genes involved in B cell activation, proliferation, and differentiation [206]. For example, in the germinal center reaction, where B cells undergo somatic hypermutation and class switching to produce high‐affinity antibodies, m6A modifications can influence the expression of genes encoding activation‐induced cytidine deaminase (AID), which is essential for these processes [207].

RNA modifications also contribute to immune memory, which is a hallmark of the adaptive immune system. In memory T and B cells, specific RNA modification patterns may be involved in maintaining the long‐term survival and rapid recall response of these cells. Understanding the roles of RNA modifications in adaptive immunity provides new opportunities for developing immunotherapies and vaccines. Manipulating RNA modifications could potentially enhance immune responses against pathogens or tumors and modulate autoimmune reactions.

## 6 Conclusions

RNA modifications are emerging as pivotal players in the intricate regulation of gene expression, with profound implications for both health and disease. These chemical modifications, such as m6A, m5C, and Ψ, serve as dynamic modulators of RNA function, influencing diverse biological processes including RNA stability, translation, and localization. This regulatory capacity underscores their critical roles in maintaining cellular homeostasis and responding to physiological changes. In healthy organisms, RNA modifications contribute to the fine‐tuning of gene expression essential for normal development and function. They play crucial roles in embryogenesis, neurodevelopment, and immune responses. Our analysis underscores a critical duality: while precisely regulated RNA modifications are indispensable for health, their dysregulation serves as a potent driver of pathogenesis. Aberrant modification landscapes are not mere bystanders but active contributors to disease etiology. In oncology, distorted m6A patterns demonstrably rewire oncogenic pathways, fueling unchecked proliferation and metastasis. Similarly, disrupted modification networks in neurological disorders (e.g., Alzheimer's, autism) impair synaptic function and neuronal resilience. Links to cardiovascular pathologies and altered host–viral interactions in infectious diseases further highlight the broad pathological footprint of epitranscriptomic dysregulation. This compelling disease association firmly positions the RNA modification machinery—writers, erasers, readers—as a novel and highly promising frontier for therapeutic intervention. Strategies employing small molecule inhibitors or RNA‐targeting technologies offer potential to recalibrate these dysregulated pathways. By enabling rapid and reversible changes to the transcriptome, RNA modifications allow cells to adapt to environmental cues and stresses efficiently. This adaptability is vital for processes such as stem cell differentiation, neuronal plasticity, and immune cell activation. Aberrations in RNA modification patterns have been implicated in a wide range of diseases, highlighting their importance in pathology. In cancer, dysregulated m6A methylation can lead to altered cell proliferation and survival, driving tumor growth. Neurological disorders, including Alzheimer's and autism, have been associated with changes in RNA modification landscapes, affecting neuronal function and communication. CVDs and infectious diseases also show links to RNA modification dynamics, influencing disease progression and outcomes. The burgeoning field of epitranscriptomics holds tremendous potential for advancing our understanding of disease mechanisms and developing novel therapeutic strategies. Ongoing research aims to unravel the complex networks of “writers, ” “erasers, ” and “readers” that govern RNA modifications. High‐throughput sequencing technologies and advanced computational tools are being developed to map these modifications with high precision, offering insights into their context‐specific roles. Therapeutically, targeting RNA modification pathways presents a promising strategy. Small molecule inhibitors and RNA editing technologies are being explored to modulate RNA modifications, offering potential treatments for cancers, neurological disorders, and viral infections. The ability to selectively alter RNA modifications could revolutionize personalized medicine, providing tailored interventions based on individual epitranscriptomic profiles. RNA modifications are integral to the regulation of gene expression, with significant roles in both maintaining health and contributing to disease. The dynamic and reversible nature of these modifications makes them attractive targets for therapeutic intervention. As research advances, the field of RNA modifications is poised to offer novel insights and transformative approaches to diagnosing and treating a wide array of diseases, heralding a new era in molecular medicine. In this review, we also provide an overview of the PTMs involved in the m6A process, such as ubiquitination, phosphorylation, SUMOylation, O‐GlcNAc, and lactylation. These modifications can trigger unique PPIs, influencing various physiological processes and diseases. Despite significant advances, challenges remain, including the complexity of modification networks and their context‐dependent effects. Current research often focuses on individual modifications, so comprehensive studies on their interactions are needed. The specificity and dynamics of these modifications in different cellular contexts are not fully understood, highlighting the need for technologies that can detect and quantify them with high sensitivity and precision. Future research should encourage integrated studies using multi‐omics approaches, develop advanced tools for real‐time monitoring in live cells, and explore therapeutic potentials. Understanding the mechanistic roles of these modifications in regulating m6A elements is crucial. Promoting data sharing and standardization within the research community will accelerate the application and validation of new discoveries. Addressing these challenges can unlock new therapeutic strategies and deepen our understanding of m6A modifications, holding great promise for advancements in treating cancer and other diseases.

## Author Contributions

S.L. and P.L. drafted the manuscript. S.L. drew the figures. X.L. conceived the review and revised the manuscript. J.F., Y.L., and J.T. reviewed the manuscript. All authors read and approved the final manuscript.

## Ethics Statement

The authors have nothing to report.

## Conflicts of Interest

The authors declare no conflicts of interest.

## Data Availability

The authors have nothing to report.

## References

[1] I. A. Roundtree , M. E. Evans , T. Pan , and C. He , “Dynamic RNA Modifications in Gene Expression Regulation, ” Cell 169, no. 7 (2017): 1187–1200. 28622506 10.1016/j.cell.2017.05.045 PMC5657247

[2] A. G. Torres , E. Batlle , and L. Ribas de Pouplana , “Role of tRNA Modifications in human Diseases, ” Trends in Molecular Medicine 20, no. 6 (2014): 306–314. 24581449 10.1016/j.molmed.2014.01.008

[3] W. E. Cohn , “Pseudouridine, a Carbon‐carbon Linked Ribonucleoside in Ribonucleic Acids: Isolation, Structure, and Chemical Characteristics, ” Journal of Biological Chemistry 235 (1960): 1488–1498. 13811056

[4] R. W. Holley , J. Apgar , G. A. Everett , et al., “Structure of a Ribonucleic Acid,” Science 147, no. 3664 (1965): 1462–1465. 14263761 10.1126/science.147.3664.1462

[5] R. Desrosiers , K. Friderici , F. Rottman , “Identification of Methylated Nucleosides in Messenger RNA From Novikoff Hepatoma Cells,” Proceedings of the National Academy of Sciences of the United States of America 71, no. 10 (1974): 3971–3975. 4372599 10.1073/pnas.71.10.3971 PMC434308

[6] T. Selmi , S. Hussain , S. Dietmann , et al., “Sequence‐ and Structure‐specific Cytosine‐5 mRNA Methylation by NSUN6, ” Nucleic Acids Res 49, no. 2 (2021): 1006–1022. 33330931 10.1093/nar/gkaa1193 PMC7826283

[7] K. E. Bohnsack , C. Höbartner , and M. T. Bohnsack , “Eukaryotic 5‐methylcytosine (m^5^C) RNA Methyltransferases: Mechanisms, Cellular Functions, and Links to Disease, ” Genes 10, no. 2 (2019): 102. 30704115 10.3390/genes10020102 PMC6409601

[8] Y. Y. Wang , Y. Tian , Y. Z. Li , et al., “The Role of m5C Methyltransferases in Cardiovascular Diseases, ” Frontiers in Cardiovascular Medicine 10 (2023): 1225014. 37476573 10.3389/fcvm.2023.1225014 PMC10354557

[9] S. Oerum , C. Dégut , P. Barraud , and C. Tisné , “m1A Post‐Transcriptional Modification in tRNAs, ” Biomolecules 7, no. 1 (2017): 20. 28230814 10.3390/biom7010020 PMC5372732

[10] X. Zhang , W. Y. Zhu , S. Y. Shen , J. H. Shen , and X. D. Chen , “Biological Roles of RNA m7G Modification and Its Implications in Cancer, ” Biology Direct 18, no. 1 (2023): 58. 37710294 10.1186/s13062-023-00414-5 PMC10500781

[11] Y. Li , Y. Yi , X. Gao , et al., “2'‐O‐methylation at Internal Sites on mRNA Promotes mRNA Stability, ” Molecular Cell 84, no. 12 (2024): 2320–2336.e6. 38906115 10.1016/j.molcel.2024.04.011 PMC11196006

[12] D. Wiener and S. Schwartz , “The Epitranscriptome Beyond m6A, ” Nature Reviews Genetics 22, no. 2 (2021): 119–131. 10.1038/s41576-020-00295-8 33188361

[13] X. Deng , R. Su , H. Weng , H. Huang , Z. Li , and J. Chen , “RNA N6‐methyladenosine Modification in Cancers: Current Status and Perspectives, ” Cell Research 28, no. 5 (2018): 507–517. 29686311 10.1038/s41422-018-0034-6 PMC5951805

[14] J. Liu , Y. Yue , D. Han , et al., “A METTL3‐METTL14 Complex Mediates Mammalian Nuclear RNA N6‐adenosine Methylation, ” Nature Chemical Biology 10, no. 2 (2014): 93–95. 24316715 10.1038/nchembio.1432 PMC3911877

[15] X. L. Ping , B. F. Sun , L. Wang , et al., “Mammalian WTAP Is a Regulatory Subunit of the RNA N6‐methyladenosine Methyltransferase, ” Cell Research 24, no. 2 (2014): 177–189. 24407421 10.1038/cr.2014.3 PMC3915904

[16] B. I. Fedeles , V. Singh , J. C. Delaney , D. Li , and J. M. Essigmann , “The AlkB Family of Fe(II)/α‐Ketoglutarate‐dependent Dioxygenases: Repairing Nucleic Acid Alkylation Damage and Beyond, ” Journal of Biological Chemistry 290, no. 34 (2015): 20734–20742. 26152727 10.1074/jbc.R115.656462 PMC4543635

[17] X. Zhao , Y. Yang , B. F. Sun , et al., “FTO‐dependent Demethylation of N6‐methyladenosine Regulates mRNA Splicing and Is Required for Adipogenesis, ” Cell Research 24, no. 12 (2014): 1403–1419. 25412662 10.1038/cr.2014.151 PMC4260349

[18] M. Merkestein , S. Laber , F. McMurray , et al., “FTO Influences Adipogenesis by Regulating Mitotic Clonal Expansion, ” Nature Communications 6 (2015): 6792. 10.1038/ncomms7792 PMC4410642 25881961

[19] G. Zheng , J. A. Dahl , Y. Niu , et al., “ALKBH5 Is a Mammalian RNA Demethylase That Impacts RNA Metabolism and Mouse Fertility, ” Molecular Cell 49, no. 1 (2013): 18–29. 23177736 10.1016/j.molcel.2012.10.015 PMC3646334

[20] X. Y. Chen , J. Zhang , and J. S. Zhu , “The Role of m6A RNA Methylation in human Cancer, ” Molecular cancer 18 (2019): 103. 31142332 10.1186/s12943-019-1033-z PMC6540575

[21] G. Casella , D. Tsitsipatis , K. Abdelmohsen , and M. Gorospe , “mRNA Methylation in Cell Senescence, ” Wiley Interdiscip Rev RNA 10, no. 6 (2019): e1547. 31144457 10.1002/wrna.1547 PMC8474013

[22] X. Wang , Z. Lu , A. Gomez , et al., “m6A‐dependent Regulation of Messenger RNA Stability, ” Nature 505, no. 7481 (2014): 117–120. 24284625 10.1038/nature12730 PMC3877715

[23] C. Xu , X. Wang , K. Liu , et al., “Structural Basis for Selective Binding of m6A RNA by the YTHDC1 YTH Domain, ” Nature Chemical Biology 10, no. 11 (2014): 927–929. 25242552 10.1038/nchembio.1654

[24] S. Müller , M. Glaß , A. K. Singh , et al., “IGF2BP1 promotes SRF‐dependent Transcription in Cancer in a m6A‐ and miRNA‐dependent Manner, ” Nucleic Acids Res. 47, no. 1 (2019): 375–390. 30371874 10.1093/nar/gky1012 PMC6326824

[25] S. Wang , B. Chim , Y. Su , et al., “Enhancement of LIN28B‐induced Hematopoietic Reprogramming by IGF2BP3, ” Genes & development 33, no. 15‐16 (2019): 1048–1068. 31221665 10.1101/gad.325100.119 PMC6672051

[26] T. Li , P. S. Hu , Z. Zuo , et al., “METTL3 facilitates Tumor Progression via an m6A‐IGF2BP2‐dependent Mechanism in Colorectal Carcinoma, ” Molecular cancer 18 (2019): 112. 31230592 10.1186/s12943-019-1038-7 PMC6589893

[27] B. Chen , Y. Li , R. Song , C. Xue , and F. Xu , “Functions of RNA N6‐methyladenosine Modification in Cancer Progression, ” Molecular Biology Reports 46, no. 1 (2019): 1383–1391. 30788764 10.1007/s11033-018-4471-6

[28] Z. Bi , Y. Liu , Y. Zhao , et al., “A Dynamic Reversible RNA N6 ‐methyladenosine Modification: Current Status and Perspectives, ” Journal of Cellular Physiology 234, no. 6 (2019): 7948–7956. 30644095 10.1002/jcp.28014

[29] N. Pinello , S. Sun , and J. J. L. Wong , “Aberrant Expression of Enzymes Regulating m6A mRNA Methylation: Implication in Cancer, ” Cancer Biol Med 15, no. 4 (2018): 323–334. 30766746 10.20892/j.issn.2095-3941.2018.0365 PMC6372906

[30] S. Zaccara , R. J. Ries , and S. R. Jaffrey , “Reading, Writing and Erasing mRNA Methylation, ” Nature Reviews Molecular Cell Biology 20, no. 10 (2019): 608–624. 31520073 10.1038/s41580-019-0168-5

[31] R. Desrosiers , K. Friderici , and F. Rottman , “Identification of Methylated Nucleosides in Messenger RNA From Novikoff Hepatoma Cells, ” PNAS 71, no. 10 (1974): 3971–3975. 4372599 10.1073/pnas.71.10.3971 PMC434308

[32] P. Wang , K. A. Doxtader , and Y. Nam , “Structural Basis for Cooperative Function of Mettl3 and Mettl14 Methyltransferases, ” Molecular Cell 63, no. 2 (2016): 306–317. 27373337 10.1016/j.molcel.2016.05.041 PMC4958592

[33] S. Schwartz , M. R. Mumbach , M. Jovanovic , et al., “Perturbation of m6A Writers Reveals Two Distinct Classes of mRNA Methylation at Internal and 5' sites, ” Cell reports 8, no. 1 (2014): 284–296. 24981863 10.1016/j.celrep.2014.05.048 PMC4142486

[34] Y. Yue , J. Liu , X. Cui , et al., “VIRMA Mediates Preferential m6A mRNA Methylation in 3'UTR and near Stop Codon and Associates With Alternative Polyadenylation, ” Cell Discovery 4 (2018): 10. 29507755 10.1038/s41421-018-0019-0 PMC5826926

[35] P. Knuckles , T. Lence , I. U. Haussmann , et al., “Zc3h13/Flacc Is Required for Adenosine Methylation by Bridging the mRNA‐binding Factor Rbm15/Spenito to the m6A Machinery Component Wtap/Fl(2)D, ” Genes & development 32, no. 5‐6 (2018): 415–429. 29535189 10.1101/gad.309146.117 PMC5900714

[36] D. P. Patil , C. K. Chen , B. F. Pickering , et al., “A RNA Methylation Promotes XIST‐mediated Transcriptional Repression, ” Nature 537, no. 7620 (2016): 369–373. 27602518 10.1038/nature19342 PMC5509218

[37] I. Barbieri , K. Tzelepis , L. Pandolfini , et al., “Promoter‐bound METTL3 Maintains Myeloid Leukaemia via m6A‐dependent Translation Control, ” Nature 552, no. 7683 (2017): 126–131. 29186125 10.1038/nature24678 PMC6217924

[38] A. Bertero , S. Brown , P. Madrigal , et al., “The SMAD2/3 Interactome Reveals That TGFβ Controls m6A mRNA Methylation in Pluripotency, ” Nature 555, no. 7695 (2018): 256–259. 29489750 10.1038/nature25784 PMC5951268

[39] F. Yu , J. Wei , X. Cui , et al., “Post‐translational Modification of RNA m6A Demethylase ALKBH5 Regulates ROS‐induced DNA Damage Response, ” Nucleic Acids Res. 49, no. 10 (2021): 5779–5797. 34048572 10.1093/nar/gkab415 PMC8191756

[40] G. Zhu , L. Jin , W. Sun , S. Wang , and N. Liu , “Proteomics of Post‐translational Modifications in Colorectal Cancer: Discovery of New Biomarkers, ” Biochim Biophys Acta Rev Cancer 1877, no. 4 (2022): 188735. 35577141 10.1016/j.bbcan.2022.188735

[41] J. R. Johnson , D. C. Crosby , J. F. Hultquist , et al., “Global Post‐translational Modification Profiling of HIV‐1‐infected Cells Reveals Mechanisms of Host Cellular Pathway Remodeling, ” Cell reports 39, no. 2 (2022): 110690. 35417684 10.1016/j.celrep.2022.110690 PMC9429972

[42] P. M. V. Shetty , A. Y. Rangrez , and N. Frey , “SUMO Proteins in the Cardiovascular System: Friend or Foe?, ” Journal of Biomedical Science 27, no. 1 (2020): 98. 33099299 10.1186/s12929-020-00689-0 PMC7585181

[43] C. Pohl and I. Dikic , “Cellular Quality Control by the Ubiquitin‐proteasome System and Autophagy, ” Science 366, no. 6467 (2019): 818–822. 31727826 10.1126/science.aax3769

[44] Z. C. Zeng , Q. Pan , Y. M. Sun , et al., “METTL3 protects METTL14 From STUB1‐mediated Degradation to Maintain m6A Homeostasis, ” Embo Reports (2023): e55762, 10.15252/embr.202255762. n/a(n/a). 36597993 PMC9986817

[45] Y. Wang , Y. Li , J. I. Toth , M. D. Petroski , Z. Zhang , and J. C. Zhao , “N6‐methyladenosine Modification Destabilizes Developmental Regulators in Embryonic Stem Cells, ” Nature Cell Biology 16, no. 2 (2014): 191–198. 24394384 10.1038/ncb2902 PMC4640932

[46] Y. Yang , X. Fan , M. Mao , “Extensive Translation of Circular RNAs Driven by N6‐methyladenosine, ” Cell Research 27, no. 5 (2017): 626–641. 28281539 10.1038/cr.2017.31 PMC5520850

[47] L. Dong , D. Lu , R. Chen , et al., “Proteogenomic Characterization Identifies Clinically Relevant Subgroups of Intrahepatic Cholangiocarcinoma, ” Cancer Cell 40, no. 1 (2022): 70–87.e15. 34971568 10.1016/j.ccell.2021.12.006

[48] J. Wei , B. T. Harada , D. Lu , et al., “HRD1‐mediated METTL14 Degradation Regulates m6A mRNA Modification to Suppress ER Proteotoxic Liver Disease, ” Molecular Cell 81, no. 24 (2021): 5052–5065.e6. 34847358 10.1016/j.molcel.2021.10.028 PMC8751812

[49] Z. Liu , K. Wu , S. Gu , et al., “A Methyltransferase‐Like 14/miR‐99a‐5p/Tribble 2 Positive Feedback Circuit Promotes Cancer Stem Cell Persistence and Radioresistance via Histone Deacetylase 2‐mediated Epigenetic Modulation in Esophageal Squamous Cell Carcinoma, ” Clinical and translational medicine 11, no. 9 (2021): e545. 34586732 10.1002/ctm2.545 PMC8441142

[50] J. A. Harrigan , X. Jacq , N. M. Martin , and S. P. Jackson , “Deubiquitylating Enzymes and Drug Discovery: Emerging Opportunities, ” Nat Rev Drug Discovery 17, no. 1 (2018): 57–78. 28959952 10.1038/nrd.2017.152 PMC7097658

[51] J. Huang , W. Zhou , C. Hao , Q. He , and X. Tu , “The Feedback Loop of METTL14 and USP38 Regulates Cell Migration, Invasion and EMT as Well as Metastasis in Bladder Cancer, ” PLos Genet 18, no. 10 (2022): e1010366. 36288387 10.1371/journal.pgen.1010366 PMC9605029

[52] P. Lu , Y. Xu , S. Z. yong , “De‐ubiquitination of p300 by USP12 Critically Enhances METTL3 Expression and Ang II‐induced Cardiac Hypertrophy, ” Experimental Cell Research 406, no. 1 (2021): 112761. 34339675 10.1016/j.yexcr.2021.112761

[53] X. Xiao , J. Shi , C. He , et al., “ERK and USP5 Govern PD‐1 Homeostasis via Deubiquitination to Modulate Tumor Immunotherapy, ” Nature Communications 14, no. 1 (2023): 2859. 10.1038/s41467-023-38605-3 PMC10199079 37208329

[54] H. L. Sun , A. C. Zhu , Y. Gao , et al., “Stabilization of ERK‐Phosphorylated METTL3 by USP5 Increases m6A Methylation, ” Molecular Cell 80, no. 4 (2020): 633. 33217317 10.1016/j.molcel.2020.10.026 PMC7720844

[55] B. G. Beatty , S. Qi , M. Pienkowska , et al., “Chromosomal Localization of Phospholipase A2 Activating Protein, an Ets2 Target Gene, ” Genomics 62, no. 3 (1999): 529–532. to 9p21.. 10644453 10.1006/geno.1999.5999

[56] J. Z. Roberts , N. Crawford , and D. B. Longley , “The Role of Ubiquitination in Apoptosis and Necroptosis, ” Cell Death and Differentiation 29, no. 2 (2022): 272–284. 34912054 10.1038/s41418-021-00922-9 PMC8817035

[57] Z. Shen , L. Gu , Y. Liu , et al., “PLAA Suppresses Ovarian Cancer Metastasis via METTL3‐mediated m6A Modification of TRPC3 mRNA, ” Oncogene 41, no. 35 (2022): 4145–4158. 35869392 10.1038/s41388-022-02411-w PMC9418004

[58] A. P. Koivisto , M. G. Belvisi , R. Gaudet , and A. Szallasi , “Advances in TRP Channel Drug Discovery: From Target Validation to Clinical Studies, ” Nat Rev Drug Discovery 21, no. 1 (2022): 41–59. 34526696 10.1038/s41573-021-00268-4 PMC8442523

[59] .C. D. E. .C. Signaling Cell 131, no. 6 (2007): 1047–1058. 18083096 10.1016/j.cell.2007.11.028

[60] L. Liao , Y. He , S. J. Li , et al., “Anti‐HIV Drug Elvitegravir Suppresses Cancer Metastasis via Increased Proteasomal Degradation of m6A Methyltransferase METTL3, ” Cancer Research 82, no. 13 (2022): 2444–2457. 35507004 10.1158/0008-5472.CAN-21-4124

[61] J. Nedergaard , B. Cannon . Chapter 9 ‐ Brown Adipose Tissue as a Heat‐producing thermoeffector. In: Romanovsky AA , ed., ed. Handbook of Clinical Neurology. Vol 156. Thermoregulation: From Basic Neuroscience to Clinical Neurology Part I. Elsevier; 2018:137–152. 30454587 10.1016/B978-0-444-63912-7.00009-6

[62] X. Tao , R. Du , S. Guo , et al., “PGE2‐EP3 axis Promotes Brown Adipose Tissue Formation Through Stabilization of WTAP RNA Methyltransferase, ” Embo Journal 41, no. 16 (2022): e110439. 35781818 10.15252/embj.2021110439 PMC9379545

[63] T. Gerken , C. A. Girard , Y. C. L. Tung , et al., “The Obesity‐Associated FTO Gene Encodes a 2‐Oxoglutarate–Dependent Nucleic Acid Demethylase, ” Science 318, no. 5855 (2007): 1469–1472. 17991826 10.1126/science.1151710 PMC2668859

[64] M. A. Russell and .M. NG , “Conditional Expression of the FTO Gene Product in Rat INS‐1 Cells Reveals Its Rapid Turnover and a Role in the Profile of Glucose‐induced Insulin Secretion, ” Clin Sci 120, no. 9 (2011): 403–413. 10.1042/CS20100416 21070190

[65] P. Gulati , E. Avezov , M. Ma , et al., “Fat Mass and Obesity‐related (FTO) Shuttles Between the Nucleus and Cytoplasm, ” Bioscience Reports 34, no. 5 (2014): e00144. 25242086 10.1042/BSR20140111 PMC4206862

[66] T. Zhu , X. L. H. Yong , D. Xia , J. Widagdo , and V. Anggono , “Ubiquitination Regulates the Proteasomal Degradation and Nuclear Translocation of the Fat Mass and Obesity‐Associated (FTO) Protein, ” Journal of Molecular Biology 430, no. 3 (2018): 363–371. 29237556 10.1016/j.jmb.2017.12.003

[67] Y. Li , R. Su , X. Deng , Y. Chen , and J. Chen , “FTO in Cancer: Functions, Molecular Mechanisms, and Therapeutic Implications, ” Trends in cancer 8, no. 7 (2022): 598–614. 35346615 10.1016/j.trecan.2022.02.010

[68] Y. Niu , Z. Lin , A. Wan , “RNA N6‐methyladenosine Demethylase FTO Promotes Breast Tumor Progression Through Inhibiting BNIP3, ” Molecular cancer 18 (2019): 46. 30922314 10.1186/s12943-019-1004-4 PMC6437932

[69] S. Yang , J. Wei , Y. H. Cui , et al., “m6A mRNA Demethylase FTO Regulates Melanoma Tumorigenicity and Response to anti‐PD‐1 Blockade, ” Nature Communications 10 (2019): 2782. 10.1038/s41467-019-10669-0 PMC6592937 31239444

[70] D. Zou , L. Dong , C. Li , Z. Yin , S. Rao , and Q. Zhou , “The m6A Eraser FTO Facilitates Proliferation and Migration of human Cervical Cancer Cells, ” Cancer cell international 19 (2019): 321. 31827395 10.1186/s12935-019-1045-1 PMC6888952

[71] D. Y. Ruan , T. Li , Y. N. Wang , et al., “FTO Downregulation Mediated by Hypoxia Facilitates Colorectal Cancer Metastasis, ” Oncogene 40, no. 33 (2021): 5168–5181. 34218271 10.1038/s41388-021-01916-0 PMC8376648

[72] X. Bian , D. Shi , K. Xing , et al., “AMD1 upregulates Hepatocellular Carcinoma Cells Stemness by FTO Mediated mRNA Demethylation, ” Clinical and translational medicine 11, no. 3 (2021): e352. 33783988 10.1002/ctm2.352 PMC7989706

[73] Stroke. The Lancet 2017;389(10069):641–654. 10.1016/S0140-6736(16)30962-X 27637676

[74] H. M. Eilken and R. H. Adams , “Dynamics of Endothelial Cell Behavior in Sprouting Angiogenesis, ” Current Opinion in Cell Biology 22, no. 5 (2010): 617–625. 20817428 10.1016/j.ceb.2010.08.010

[75] I. Geudens and H. Gerhardt , “Coordinating Cell Behaviour During Blood Vessel Formation, ” Development (Cambridge, England) 138, no. 21 (2011): 4569–4583. 21965610 10.1242/dev.062323

[76] B. Han , Y. Zhang , Y. Zhang , et al., “Novel Insight Into Circular RNA HECTD1 in Astrocyte Activation via Autophagy by Targeting MIR142‐TIPARP: Implications for Cerebral Ischemic Stroke, ” Autophagy 14, no. 7 (2018): 1164. 29938598 10.1080/15548627.2018.1458173 PMC6103660

[77] P. A. Mueller , L. Yang , M. Ubele , et al., “The Coronary Artery Disease Risk‐associated Plpp3 Gene and Its Product Lipid Phosphate Phosphatase 3 Regulate Experimental Atherosclerosis, ” Arteriosclerosis, Thrombosis, and Vascular Biology 39, no. 11 (2019): 2261–2272. 31533471 10.1161/ATVBAHA.119.313056 PMC6812632

[78] B. Li , W. Xi , Y. Bai , et al., “FTO‐dependent m6A Modification of Plpp3 in circSCMH1‐regulated Vascular Repair and Functional Recovery Following Stroke, ” Nature Communications 14 (2023): 489. 10.1038/s41467-023-36008-y PMC9886939 36717587

[79] C. Liu , Y. Li , C. Dong , L. Qu , and Y. Zuo , “E6E7 regulates the HK2 Expression in Cervical Cancer via GSK3β/FTO Signal, ” Archives of Biochemistry and Biophysics 729 (2022): 109389. 36075458 10.1016/j.abb.2022.109389

[80] Z. Zhang , Q. Gao , and S. Wang , “Kinase GSK3β Functions as a Suppressor in Colorectal Carcinoma Through the FTO‐mediated MZF1/c‐Myc Axis, ” Journal of Cellular and Molecular Medicine 25, no. 5 (2021): 2655–2665. 33533172 10.1111/jcmm.16291 PMC7933972

[81] C. Wen , M. Lan , X. Tan , et al., “GSK3β Exacerbates Myocardial Ischemia/Reperfusion Injury by Inhibiting Myc, ” Oxid Med Cell Longev 2022 (2022): 2588891. 35528516 10.1155/2022/2588891 PMC9076327

[82] J. Pan , L. Xu , and H. Pan , “Development and Validation of an m6A RNA Methylation Regulator‐Based Signature for Prognostic Prediction in Cervical Squamous Cell Carcinoma, ” Frontiers in oncology 10 (2020): 1444. 32974164 10.3389/fonc.2020.01444 PMC7472601

[83] M. Hirayama , F. Y. Wei , T. Chujo , et al., “FTO Demethylates Cyclin D1 mRNA and Controls Cell‐Cycle Progression, ” Cell reports 31, no. 1 (2020): 107464. 32268083 10.1016/j.celrep.2020.03.028

[84] FTO regulates the chemo‐radiotherapy resistance of cervical squamous cell carcinoma (CSCC) by targeting β‐catenin through mRNA demethylation—Zhou ‐ 2018 ‐ Molecular Carcinogenesis—Wiley Online Library. Accessed March 20, 2023. https://onlinelibrary.wiley.com/doi/10.1002/mc.22782 10.1002/mc.22782 29315835

[85] W. Song , K. Yang , J. Luo , Z. Gao , and Y. Gao , “Dysregulation of USP18/FTO/PYCR1 Signaling Network Promotes Bladder Cancer Development and Progression, ” Aging 13, no. 3 (2021): 3909–3925. 33461172 10.18632/aging.202359 PMC7906198

[86] S. C. Trewick , T. F. Henshaw , R. P. Hausinger , T. Lindahl , and B. Sedgwick , “Oxidative Demethylation by Escherichia coli AlkB Directly Reverts DNA Base Damage, ” Nature 419, no. 6903 (2002): 174–178. 12226667 10.1038/nature00908

[87] K. Tsujikawa , K. Koike , K. Kitae , et al., “Expression and Sub‐cellular Localization of human ABH family Molecules, ” Journal of Cellular and Molecular Medicine 11, no. 5 (2007): 1105–1116. 17979886 10.1111/j.1582-4934.2007.00094.x PMC4401260

[88] G. Chang , G. S. Xie , L. Ma , P. Li , L. Li , and H. T. Richard , “USP36 promotes Tumorigenesis and Drug Sensitivity of Glioblastoma by Deubiquitinating and Stabilizing ALKBH5, ” Neuro‐Oncology: noac238, 10.1093/neuonc/noac238. Published online October 14, 2022. PMC10158114 36239338

[89] A. Wang , H. Huang , J. H. Shi , et al., “USP47 inhibits m6A‐dependent c‐Myc Translation to Maintain Regulatory T Cell Metabolic and Functional Homeostasis, ” Journal of Clinical Investigation 133, no. 23: e169365. 10.1172/JCI169365 PMC10688989 37788092

[90] Y. Chen , R. Wu , W. Chen , et al., “Curcumin Prevents Obesity by Targeting TRAF4‐induced Ubiquitylation in m6A‐dependent Manner, ” Embo Reports 22, no. 5 (2021): e52146. 33880847 10.15252/embr.202052146 PMC8097347

[91] S. Zhang , X. Guan , W. Liu , et al., “YTHDF1 alleviates Sepsis by Upregulating WWP1 to Induce NLRP3 Ubiquitination and Inhibit Caspase‐1‐dependent Pyroptosis, ” Cell Death Discov 8 (2022): 244. 35508474 10.1038/s41420-022-00872-2 PMC9068740

[92] F. Xu , J. Li , M. Ni , et al., “FBW7 suppresses Ovarian Cancer Development by Targeting the N6‐methyladenosine Binding Protein YTHDF2, ” Molecular cancer 20 (2021): 45. 33658012 10.1186/s12943-021-01340-8 PMC7927415

[93] F. A. Barr , P. R. Elliott , and U. Gruneberg , “Protein Phosphatases and the Regulation of Mitosis, ” Journal of Cell Science 124, no. 14 (2011): 2323–2334. 21709074 10.1242/jcs.087106

[94] Q. Fei , Z. Zou , I. A. Roundtree , H. L. Sun , and C. He , “YTHDF2 promotes Mitotic Entry and Is Regulated by Cell Cycle Mediators, ” Plos Biology 18, no. 4 (2020): e3000664. 32267835 10.1371/journal.pbio.3000664 PMC7170294

[95] J. Paris , M. Morgan , J. Campos , et al., “Targeting the RNA m6A Reader YTHDF2 Selectively Compromises Cancer Stem Cells in Acute Myeloid Leukemia, ” Cell Stem Cell 25, no. 1 (2019): 137–148.e6. 31031138 10.1016/j.stem.2019.03.021 PMC6617387

[96] Z. R , N. W , Q. C , “A Functional Loop Between YTH Domain family Protein YTHDF3 Mediated m6A Modification and Phosphofructokinase PFKL in Glycolysis of Hepatocellular Carcinoma, ” J Exp Clin Cancer Res CR 41, no. 1 (2022), 10.1186/s13046-022-02538-4. PMC9724358 36471428

[97] X. T. Lin , H. Q. Yu , L. Fang , et al., “Elevated FBXO45 Promotes Liver Tumorigenesis Through Enhancing IGF2BP1 Ubiquitination and Subsequent PLK1 Upregulation, ” Elife 10 (2021): e70715. 34779401 10.7554/eLife.70715 PMC8641947

[98] L. S. Kristensen , M. S. Andersen , L. V. W. Stagsted , K. K. Ebbesen , T. B. Hansen , and J. Kjems , “The Biogenesis, Biology and Characterization of Circular RNAs, ” Nature Reviews Genetics 20, no. 11 (2019): 675–691. 10.1038/s41576-019-0158-7 31395983

[99] B. Li , L. Zhu , C. Lu , et al., “circNDUFB2 inhibits Non‐small Cell Lung Cancer Progression via Destabilizing IGF2BPs and Activating Anti‐tumor Immunity, ” Nature Communications 12, no. 1 (2021): 295. 10.1038/s41467-020-20527-z PMC7804955 33436560

[100] B. Yao , Q. Zhang , Z. Yang , et al., “CircEZH2/miR‐133b/IGF2BP2 Aggravates Colorectal Cancer Progression via Enhancing the Stability of m6A‐modified CREB1 mRNA, ” Molecular cancer 21 (2022): 140. 35773744 10.1186/s12943-022-01608-7 PMC9245290

[101] M. J. Watson , P. L. Berger , K. Banerjee , et al., “Aberrant CREB1 Activation in Prostate Cancer Disrupts Normal Prostate Luminal Cell Differentiation, ” Oncogene 40, no. 18 (2021): 3260–3272. 33846571 10.1038/s41388-021-01772-y PMC10760404

[102] K. M. Sakamoto and D. A. Frank , “CREB in the Pathophysiology of Cancer: Implications for Targeting Transcription Factors for Cancer Therapy, ” Clin Cancer Res Off J Am Assoc Cancer Res 15, no. 8 (2009): 2583–2587. 10.1158/1078-0432.CCR-08-1137 PMC2883446 19351775

[103] J. K. Park , S. H. Park , K. So , I. H. Bae , Y. D. Yoo , and H. D. Um , “ICAM‐3 Enhances the Migratory and Invasive Potential of human Non‐small Cell Lung Cancer Cells by Inducing MMP‐2 and MMP‐9 via Akt and CREB, ” International Journal of Oncology 36, no. 1 (2010): 181–192. 19956847

[104] D. B. Shankar , J. C. Cheng , K. Kinjo , et al., “The Role of CREB as a Proto‐oncogene in Hematopoiesis and in Acute Myeloid Leukemia, ” Cancer Cell 7, no. 4 (2005): 351–362. 15837624 10.1016/j.ccr.2005.02.018

[105] Cell cycle regulation of cyclin A gene expression by the cyclic AMP‐responsive transcription factors CREB and CREM—PubMed. Accessed March 28, 2023. https://pubmed.ncbi.nlm.nih.gov/7760825/ 10.1128/mcb.15.6.3301 PMC230563 7760825

[106] Y. Wang , J. H. Lu , Q. N. Wu , et al., “LncRNA LINRIS Stabilizes IGF2BP2 and Promotes the Aerobic Glycolysis in Colorectal Cancer, ” Molecular cancer 18 (2019): 174. 31791342 10.1186/s12943-019-1105-0 PMC6886219

[107] L. D. Harris , J. Le Pen , N. Scholz , “The Deubiquitinase TRABID Stabilizes the K29/K48‐specific E3 Ubiquitin Ligase HECTD1, ” Journal of Biological Chemistry 296 (2021): 100246. 33853758 10.1074/jbc.RA120.015162 PMC7948964

[108] A. Y. Rangrez , A. Borlepawar , N. Schmiedel , et al., “The E3 Ubiquitin Ligase HectD3 Attenuates Cardiac Hypertrophy and Inflammation in Mice, ” Communications Biology 3, no. 1 (2020): 562. 33037313 10.1038/s42003-020-01289-2 PMC7547098

[109] Z. Pan , R. Zhao , B. Li , et al., “EWSR1‐induced circNEIL3 Promotes Glioma Progression and Exosome‐mediated Macrophage Immunosuppressive Polarization via Stabilizing IGF2BP3, ” Molecular cancer 21 (2022): 16. 35031058 10.1186/s12943-021-01485-6 PMC8759291

[110] S. Kapoor , “IMP3: A New and Important Biomarker of Systemic Malignancies, ” Clin Cancer Res Off J Am Assoc Cancer Res 14, no. 17 (2008): 5640. author reply 5640–5641. 10.1158/1078-0432.CCR-08-0813 18765560

[111] B. Liao , Y. Hu , and G. Brewer , “RNA‐binding Protein Insulin‐Like Growth Factor mRNA‐binding Protein 3 (IMP‐3) Promotes Cell Survival via Insulin‐Like Growth Factor II Signaling After Ionizing Radiation, ” Journal of Biological Chemistry 286, no. 36 (2011): 31145–31152. 21757716 10.1074/jbc.M111.263913 PMC3173139

[112] C. Jia , H. Tang , Y. Yang , et al., “Ubiquitination of IGF2BP3 by E3 Ligase MKRN2 Regulates the Proliferation and Migration of human Neuroblastoma SHSY5Y Cells, ” Biochemical and Biophysical Research Communications 529, no. 1 (2020): 43–50. 32560817 10.1016/j.bbrc.2020.05.112

[113] Y. Shuai , Z. Ma , W. Liu , et al., “TEAD4 modulated LncRNA MNX1‐AS1 Contributes to Gastric Cancer Progression Partly Through Suppressing BTG2 and Activating BCL2, ” Molecular cancer 19, no. 1 (2020): 6. 31924214 10.1186/s12943-019-1104-1 PMC6953272

[114] Q. N. Wu , X. J. Luo , J. Liu , et al., “MYC‐Activated LncRNA MNX1‐AS1 Promotes the Progression of Colorectal Cancer by Stabilizing YB1, ” Cancer Research 81, no. 10 (2021): 2636–2650. 33782099 10.1158/0008-5472.CAN-20-3747

[115] F. Li , Q. Chen , H. Xue , L. Zhang , K. Wang , and F. Shen , “LncRNA MNX1‐AS1 Promotes Progression of Intrahepatic Cholangiocarcinoma Through the MNX1/Hippo Axis, ” Cell death & disease 11, no. 10 (2020): 894. 33093444 10.1038/s41419-020-03029-0 PMC7581777

[116] S. Liu , H. Li , Y. Zhu , et al., “LncRNA MNX1‐AS1 Sustains Inactivation of Hippo Pathway Through a Positive Feedback Loop With USP16/IGF2BP3 Axis in Gallbladder Cancer, ” Cancer Letters 547 (2022): 215862. 35953000 10.1016/j.canlet.2022.215862

[117] R. J. J. Jansens , R. Verhamme , A. H. Mirza , et al., “Alphaherpesvirus US3 Protein‐mediated Inhibition of the m6A mRNA Methyltransferase Complex, ” Cell reports 40, no. 3 (2022): 111107. 35858564 10.1016/j.celrep.2022.111107 PMC9347262

[118] R. Roskoski , “ERK1/2 MAP Kinases: Structure, Function, and Regulation, ” Pharmacological Research 66, no. 2 (2012): 105–143. 22569528 10.1016/j.phrs.2012.04.005

[119] H. L. Sun , A. C. Zhu , Y. Gao , et al., “Stabilization of ERK‐Phosphorylated METTL3 by USP5 Increases m6A Methylation, ” Molecular Cell 80, no. 4 (2020): 633–647. 33217317 10.1016/j.molcel.2020.10.026 PMC7720844

[120] J. H. J. Hoeijmakers , “DNA Damage, Aging, and Cancer, ” New England Journal of Medicine 361, no. 15 (2009): 1475–1485. 19812404 10.1056/NEJMra0804615

[121] M. R. Lieber , “The Mechanism of Double‐strand DNA Break Repair by the Nonhomologous DNA End‐joining Pathway, ” Annual Review of Biochemistry 79 (2010): 181–211. 10.1146/annurev.biochem.052308.093131 PMC3079308 20192759

[122] W. D. Wright , S. S. Shah , and W. D. Heyer , “Homologous Recombination and the Repair of DNA Double‐strand Breaks, ” Journal of Biological Chemistry 293, no. 27 (2018): 10524–10535. 29599286 10.1074/jbc.TM118.000372 PMC6036207

[123] S. P. Jackson and J. Bartek , “The DNA‐damage Response in human Biology and Disease, ” Nature 461, no. 7267 (2009): 1071–1078. 19847258 10.1038/nature08467 PMC2906700

[124] C. Zhang , L. Chen , D. Peng , et al., “METTL3 and N6‐Methyladenosine Promote Homologous Recombination‐Mediated Repair of DSBs by Modulating DNA‐RNA Hybrid Accumulation, ” Molecular Cell 79, no. 3 (2020): 425–442.e7. 32615088 10.1016/j.molcel.2020.06.017

[125] X. Li , B. Yuan , M. Lu , et al., “The Methyltransferase METTL3 Negatively Regulates Nonalcoholic Steatohepatitis (NASH) Progression, ” Nature Communications 12 (2021): 7213. 10.1038/s41467-021-27539-3 PMC8664922 34893641

[126] J. Chen , X. Wei , X. Wang , et al., “TBK1‐METTL3 axis Facilitates Antiviral Immunity, ” Cell reports 38, no. 7 (2022): 110373. 35172162 10.1016/j.celrep.2022.110373

[127] B. Ou , Y. Liu , X. Yang , X. Xu , Y. Yan , and J. Zhang , “C5aR1‐positive Neutrophils Promote Breast Cancer Glycolysis Through WTAP‐dependent m6A Methylation of ENO1, ” Cell death & disease 12, no. 8 (2021): 737. 34312368 10.1038/s41419-021-04028-5 PMC8313695

[128] M. Marcinkowski , T. Pilžys , D. Garbicz , et al., “Human and Arabidopsis Alpha‐ketoglutarate‐dependent Dioxygenase Homolog Proteins‐New Players in Important Regulatory Processes, ” Iubmb Life 72, no. 6 (2020): 1126–1144. 32207231 10.1002/iub.2276

[129] H. Tai , X. Wang , J. Zhou , et al., “Protein Kinase Cβ Activates Fat Mass and Obesity‐associated Protein by Influencing Its Ubiquitin/Proteasome Degradation, ” FASEB J Off Publ Fed Am Soc Exp Biol 31, no. 10 (2017): 4396–4406. 10.1096/fj.201601159RR 28626026

[130] K. J. Faulds , J. N. Egelston , L. J. Sedivy , et al., “Glycogen Synthase Kinase‐3 (GSK‐3) Activity Regulates mRNA Methylation in Mouse Embryonic Stem Cells, ” Journal of Biological Chemistry 293, no. 27 (2018): 10731–10743. 29777057 10.1074/jbc.RA117.001298 PMC6036209

[131] M. Marcinkowski , T. Pilžys , D. Garbicz , et al., “Calmodulin as Ca2+‐Dependent Interactor of FTO Dioxygenase, ” International Journal of Molecular Sciences 22, no. 19 (2021): 10869. 34639211 10.3390/ijms221910869 PMC8509707

[132] C. Zhang , D. Samanta , H. Lu , et al., “Hypoxia Induces the Breast Cancer Stem Cell Phenotype by HIF‐dependent and ALKBH5‐mediated M^6^A‐demethylation of NANOG mRNA, ” PNAS 113, no. 14 (2016): E2047–2056. 27001847 10.1073/pnas.1602883113 PMC4833258

[133] R. Fang , X. Chen , S. Zhang , et al., “EGFR/SRC/ERK‐stabilized YTHDF2 Promotes Cholesterol Dysregulation and Invasive Growth of Glioblastoma, ” Nature Communications 12 (2021): 177. 10.1038/s41467-020-20379-7 PMC7794382 33420027

[134] T. Tanoue , M. Adachi , T. Moriguchi , and E. Nishida , “A Conserved Docking Motif in MAP Kinases Common to Substrates, Activators and Regulators, ” Nature Cell Biology 2, no. 2 (2000): 110–116. 10655591 10.1038/35000065

[135] J. Rini and M. Anbalagan , “IGF2BP1: A Novel Binding Protein of p38 MAPK, ” Molecular and Cellular Biochemistry 435, no. 1‐2 (2017): 133–140. 28497370 10.1007/s11010-017-3062-5

[136] A. Szwed , E. Kim , and E. Jacinto , “Regulation and Metabolic Functions of mTORC1 and mTORC2, ” Physiological Reviews 101, no. 3 (2021): 1371–1426. 33599151 10.1152/physrev.00026.2020 PMC8424549

[137] P. L. Bernstein , D. J. Herrick , R. D. Prokipcak , and J. Ross , “Control of c‐myc mRNA Half‐life in Vitro by a Protein Capable of Binding to a Coding Region Stability Determinant, ” Genes & development 6, no. 4 (1992): 642–654. 1559612 10.1101/gad.6.4.642

[138] A. S. Urbanska , A. Janusz‐Kaminska , K. Switon , et al., “ZBP1 phosphorylation at Serine 181 Regulates Its Dendritic Transport and the Development of Dendritic Trees of Hippocampal Neurons, ” Scientific Reports 7, no. 1 (2017): 1876. 28500298 10.1038/s41598-017-01963-2 PMC5431813

[139] W. Han , S. Wang , Y. Qi , et al., “Targeting HOTAIRM1 Ameliorates Glioblastoma by Disrupting Mitochondrial Oxidative Phosphorylation and Serine Metabolism, ” Iscience 25, no. 8 (2022): 104823. 35992092 10.1016/j.isci.2022.104823 PMC9389257

[140] F. Bonnay , A. Veloso , V. Steinmann , et al., “Oxidative Metabolism Drives Immortalization of Neural Stem Cells During Tumorigenesis, ” Cell 182, no. 6 (2020): 1490–1507. 32916131 10.1016/j.cell.2020.07.039

[141] U. Ahmadov , D. Picard , J. Bartl , et al., “The Long Non‐coding RNA HOTAIRM1 Promotes Tumor Aggressiveness and Radiotherapy Resistance in Glioblastoma, ” Cell death & disease 12, no. 10 (2021): 885. 34584066 10.1038/s41419-021-04146-0 PMC8478910

[142] R. T. Hay , “SUMO: A History of Modification, ” Molecular Cell 18, no. 1 (2005): 1–12. 15808504 10.1016/j.molcel.2005.03.012

[143] Y. Du , G. Hou , H. Zhang , et al., “SUMOylation of the m6A‐RNA Methyltransferase METTL3 Modulates Its Function, ” Nucleic Acids Res. 46, no. 10 (2018): 5195–5208. 29506078 10.1093/nar/gky156 PMC6007514

[144] H. Li , D. Wang , B. Yi , et al., “SUMOylation of IGF2BP2 Promotes Vasculogenic Mimicry of Glioma via Regulating OIP5‐AS1/miR‐495‐3p Axis, ” Int J Biol Sci 17, no. 11 (2021): 2912–2930. 34345216 10.7150/ijbs.58035 PMC8326132

[145] G. Hou , X. Zhao , L. Li , et al., “SUMOylation of YTHDF2 Promotes mRNA Degradation and Cancer Progression by Increasing Its Binding Affinity With m6A‐modified mRNAs, ” Nucleic Acids Res. 49, no. 5 (2021): 2859–2877. 33577677 10.1093/nar/gkab065 PMC7969013

[146] W. Yang , L. Wang , G. Roehn , et al., “Small Ubiquitin‐Like Modifier 1–3 Is Activated in human Astrocytic Brain Tumors and Is Required for Glioblastoma Cell Survival, ” Cancer Science 104, no. 1 (2013): 70–77. 23078246 10.1111/cas.12047 PMC3608476

[147] Ubc9 promotes invasion and metastasis of lung cancer cells. Accessed March 22, 2023. https://www.spandidos‐publications.com/or/29/4/1588 10.3892/or.2013.2268 23381475

[148] W. H. Guo , L. H. Yuan , Z. H. Xiao , D. Liu , and J. X. Zhang , “Overexpression of SUMO‐1 in Hepatocellular Carcinoma: A Latent Target for Diagnosis and Therapy of Hepatoma, ” Journal of Cancer Research and Clinical Oncology 137, no. 3 (2011): 533–541. 20502916 10.1007/s00432-010-0920-x PMC11957374

[149] W. Chien , K. L. Lee , L. W. Ding , et al., “PIAS4 is an Activator of Hypoxia Signalling via VHL Suppression During Growth of Pancreatic Cancer Cells, ” British Journal of Cancer 109, no. 7 (2013): 1795–1804. 24002598 10.1038/bjc.2013.531 PMC3790182

[150] A. Hoellein , M. Fallahi , S. Schoeffmann , et al., “Myc‐induced SUMOylation Is a Therapeutic Vulnerability for B‐cell Lymphoma, ” Blood 124, no. 13 (2014): 2081–2090. 25143484 10.1182/blood-2014-06-584524 PMC4186537

[151] J. J. Driscoll , D. Pelluru , K. Lefkimmiatis , et al., “The Sumoylation Pathway Is Dysregulated in Multiple Myeloma and Is Associated With Adverse Patient Outcome, ” Blood 115, no. 14 (2010): 2827–2834. 19965618 10.1182/blood-2009-03-211045 PMC2854429

[152] G. W. Hart , M. P. Housley , and C. Slawson , “Cycling of O‐linked Beta‐N‐acetylglucosamine on Nucleocytoplasmic Proteins, ” Nature 446, no. 7139 (2007): 1017–1022. 17460662 10.1038/nature05815

[153] X. Yang and K. Qian , “Protein O‐GlcNAcylation: Emerging Mechanisms and Functions, ” Nature Reviews Molecular Cell Biology 18, no. 7 (2017): 452–465. 28488703 10.1038/nrm.2017.22 PMC5667541

[154] Highly Efficient Enrichment of O‐GlcNAc Glycopeptides Based on Chemical Oxidation and Reversible Hydrazide Chemistry. Anal Chem. Accessed April 17, 2023. https://pubs.acs.org/doi/full/10.1021/acs.analchem.1c04031 10.1021/acs.analchem.1c04031 34846842

[155] Y. Yang , Y. Yan , J. Yin , et al., “O‐GlcNAcylation of YTHDF2 Promotes HBV‐related Hepatocellular Carcinoma Progression in an N6‐methyladenosine‐dependent Manner, ” Signal Transduct Target Ther 8 (2023): 63. 36765030 10.1038/s41392-023-01316-8 PMC9918532

[156] P. Wang , D. Xie , T. Xiao , et al., “H3K18 lactylation Promotes the Progression of Arsenite‐related Idiopathic Pulmonary Fibrosis via YTHDF1/m6A/NREP, ” Journal of Hazardous Materials 461 (2024): 132582. 37742376 10.1016/j.jhazmat.2023.132582

[157] J. Yu , P. Chai , M. Xie , et al., “Histone Lactylation Drives Oncogenesis by Facilitating m6A Reader Protein YTHDF2 Expression in Ocular Melanoma, ” Genome biology 22, no. 1 (2021): 85. 33726814 10.1186/s13059-021-02308-z PMC7962360

[158] J. Xiong , J. He , J. Zhu , et al., “Lactylation‐driven METTL3‐mediated RNA m6A Modification Promotes Immunosuppression of Tumor‐infiltrating Myeloid Cells, ” Molecular Cell 82, no. 9 (2022): 1660–1677.e10. 35320754 10.1016/j.molcel.2022.02.033

[159] L. Sun , Y. Zhang , B. Yang , et al., “Lactylation of METTL16 Promotes Cuproptosis via m6A‐modification on FDX1 mRNA in Gastric Cancer, ” Nature Communications 14, no. 1 (2023): 6523. 10.1038/s41467-023-42025-8 PMC10589265 37863889

[160] X. Chen , Y. Wang , J. N. Wang , et al., “Lactylation‐driven FTO Targets CDK2 to Aggravate Microvascular Anomalies in Diabetic Retinopathy, ” EMBO Molecular Medicine 16, no. 2 (2024): 294–318. 38297099 10.1038/s44321-024-00025-1 PMC10897304

[161] P. Y. Bhattarai , G. Kim , S. C. Lim , R. Mariappan , T. Ohn , and H. S. Choi , “METTL3 stabilization by PIN1 Promotes Breast Tumorigenesis via Enhanced m6A‐dependent Translation, ” Oncogene 42, no. 13 (2023): 1010–1023. 36755057 10.1038/s41388-023-02617-6

[162] G. Chang , G. S. Xie , L. Ma , P. Li , L. Li , and H. T. Richard , “USP36 promotes Tumorigenesis and Drug Sensitivity of Glioblastoma by Deubiquitinating and Stabilizing ALKBH5, ” Neuro‐Oncology 25, no. 5 (2023): 841–853. 36239338 10.1093/neuonc/noac238 PMC10158114

[163] K. Taketo , M. Konno , A. Asai , et al., “The Epitranscriptome m6A Writer METTL3 Promotes Chemo‐ and Radioresistance in Pancreatic Cancer Cells, ” International Journal of Oncology 52, no. 2 (2018): 621–629. 29345285 10.3892/ijo.2017.4219

[164] P. Lu , Y. Xu , Z. Y. Sheng , et al., “De‐ubiquitination of p300 by USP12 Critically Enhances METTL3 Expression and Ang II‐induced Cardiac Hypertrophy, ” Experimental Cell Research 406, no. 1 (2021): 112761. 34339675 10.1016/j.yexcr.2021.112761

[165] P. K. Brindle and M. R. Montminy , “The CREB family of Transcription Activators, ” Current opinion in genetics & development 2, no. 2 (1992): 199–204. 1386267 10.1016/s0959-437x(05)80274-6

[166] X. Liu , H. Wang , X. Zhao , et al., “Arginine Methylation of METTL14 Promotes RNA N6‐methyladenosine Modification and Endoderm Differentiation of Mouse Embryonic Stem Cells, ” Nature Communications 12 (2021): 3780. 10.1038/s41467-021-24035-6 PMC8213825 34145242

[167] S. K. Azzam , H. Alsafar , and A. A. Sajini , “FTO m6A Demethylase in Obesity and Cancer: Implications and Underlying Molecular Mechanisms, ” International Journal of Molecular Sciences 23, no. 7 (2022): 3800. 35409166 10.3390/ijms23073800 PMC8998816

[168] M. Feng , X. Xie , G. Han , et al., “YBX1 is Required for Maintaining Myeloid Leukemia Cell Survival by Regulating BCL2 Stability in an m6A‐dependent Manner, ” Blood 138, no. 1 (2021): 71–85. 33763698 10.1182/blood.2020009676 PMC8667054

[169] J. Lobo , A. L. Costa , M. Cantante , et al., “m6A RNA Modification and Its Writer/Reader VIRMA/YTHDF3 in Testicular Germ Cell Tumors: A Role in Seminoma Phenotype Maintenance, ” Journal of translational medicine 17 (2019): 79. 30866959 10.1186/s12967-019-1837-z PMC6416960

[170] S. Wang , P. Chai , R. Jia , and R. Jia , “Novel Insights on m6A RNA Methylation in Tumorigenesis: A Double‐edged Sword, ” Molecular cancer 17 (2018): 101. 30031372 10.1186/s12943-018-0847-4 PMC6054842

[171] J. Choe , S. Lin , W. Zhang , et al., “mRNA Circularization by METTL3‐eIF3h Enhances Translation and Promotes Oncogenesis, ” Nature 561, no. 7724 (2018): 556–560. 30232453 10.1038/s41586-018-0538-8 PMC6234840

[172] Y. Zhou , Z. Yin , B. Hou , et al., “Expression Profiles and Prognostic Significance of RNA N6‐methyladenosine‐related Genes in Patients With Hepatocellular Carcinoma: Evidence From Independent Datasets, ” Cancer Manag Res 11 (2019): 3921–3931. 31118805 10.2147/CMAR.S191565 PMC6503205

[173] X. Cai , X. Wang , C. Cao , et al., “HBXIP‐elevated Methyltransferase METTL3 Promotes the Progression of Breast Cancer via Inhibiting Tumor Suppressor Let‐7G, ” Cancer Letters 415 (2018): 11–19. 29174803 10.1016/j.canlet.2017.11.018

[174] Y. Wang , J. Wei , L. Feng , et al., “Aberrant m5C Hypermethylation Mediates Intrinsic Resistance to Gefitinib Through NSUN2/YBX1/QSOX1 Axis in EGFR‐mutant Non‐small‐cell Lung Cancer, ” Molecular cancer 22 (2023): 81. 37161388 10.1186/s12943-023-01780-4 PMC10169458

[175] Y. Hu , C. Chen , X. Tong , et al., “NSUN2 modified by SUMO‐2/3 Promotes Gastric Cancer Progression and Regulates mRNA m5C Methylation, ” Cell death & disease 12, no. 9 (2021): 842. 34504059 10.1038/s41419-021-04127-3 PMC8429414

[176] L. Sun , H. Zhang , and P. Gao , “Metabolic Reprogramming and Epigenetic Modifications on the Path to Cancer, ” Protein Cell 13, no. 12 (2022): 877–919. 34050894 10.1007/s13238-021-00846-7 PMC9243210

[177] S. M. Jan , A. Fahira , E. S. G. Hassan , A. S. Abdelhameed , D. Wei , and A. Wadood , “Integrative Approaches to m6A and m5C RNA Modifications in Autism Spectrum Disorder Revealing Potential Causal Variants, ” Mamm Genome Off J Int Mamm Genome Soc Published online December 30, 2024. 10.1007/s00335-024-10095-8 39738578

[178] X. Ren , Z. Feng , X. Ma , et al., “m6A/m1A/m5C‐Associated Methylation Alterations and Immune Profile in MDD, ” Molecular Neurobiology 61, no. 10 (2024): 8000–8025. 38453794 10.1007/s12035-024-04042-6 PMC11415454

[179] Y. Huang , J. Yan , Q. Li , et al., “Meclofenamic Acid Selectively Inhibits FTO Demethylation of m6A Over ALKBH5, ” Nucleic Acids Res. 43, no. 1 (2015): 373–384. 25452335 10.1093/nar/gku1276 PMC4288171

[180] Q. Cui , H. Shi , P. Ye , et al., “m6A RNA Methylation Regulates the Self‐Renewal and Tumorigenesis of Glioblastoma Stem Cells, ” Cell reports 18, no. 11 (2017): 2622–2634. 28297667 10.1016/j.celrep.2017.02.059 PMC5479356

[181] Y. Huang , R. Su , Y. Sheng , et al., “Small‐molecule Targeting of Oncogenic FTO Demethylase in Acute Myeloid Leukemia, ” Cancer Cell 35, no. 4 (2019): 677–691.e10. 30991027 10.1016/j.ccell.2019.03.006 PMC6812656

[182] T. Fukumoto , H. Zhu , T. Nacarelli , et al., “N6‐methylation of Adenosine (m6A) of FZD10 mRNA Contributes to PARP Inhibitor Resistance, ” Cancer Research 79, no. 11 (2019): 2812–2820. 30967398 10.1158/0008-5472.CAN-18-3592 PMC6548690

[183] A. Visvanathan , V. Patil , A. Arora , et al., “Essential Role of METTL3‐mediated m6A Modification in Glioma Stem‐Like Cells Maintenance and Radioresistance, ” Oncogene 37, no. 4 (2018): 522–533. 28991227 10.1038/onc.2017.351

[184] Z. Y. Liu , L. C. Lin , Z. Y. Liu , J. J. Yang , and H. Tao , “m6A epitranscriptomic and Epigenetic Crosstalk in Cardiac Fibrosis, ” Mol Ther J Am Soc Gene Ther 32, no. 4 (2024): 878–889. 10.1016/j.ymthe.2024.01.037 PMC11163196 38311850

[185] P. Zhukovsky , E. S. Tio , G. Coughlan , et al., “Genetic Influences on Brain and Cognitive Health and Their Interactions With Cardiovascular Conditions and Depression, ” Nature Communications 15, no. 1 (2024): 5207. 10.1038/s41467-024-49430-7 PMC11189393 38890310

[186] P. Krishnamurthy , E. Lambers , S. Verma , et al., “Myocardial Knockdown of mRNA‐stabilizing Protein HuR Attenuates Post‐MI Inflammatory Response and Left Ventricular Dysfunction in IL‐10‐null Mice, ” Faseb Journal 24, no. 7 (2010): 2484–2494. 20219984 10.1096/fj.09-149815 PMC2887267

[187] P. Mathiyalagan , M. Adamiak , J. Mayourian , et al., “FTO‐Dependent m6A Regulates Cardiac Function during Remodeling and Repair, ” Circulation 139, no. 4 (2019): 518–532. 29997116 10.1161/CIRCULATIONAHA.118.033794 PMC6400591

[188] T. Berulava , E. Buchholz , V. Elerdashvili , et al., “Changes in m6A RNA Methylation Contribute to Heart Failure Progression by Modulating Translation, ” European Journal of Heart Failure 22, no. 1 (2020): 54–66. 31849158 10.1002/ejhf.1672

[189] J. Shi , C. Yang , J. Zhang , et al., “NAT10 Is Involved in Cardiac Remodeling through ac4C‐Mediated Transcriptomic Regulation, ” Circulation Research 133, no. 12 (2023): 989–1002. 37955115 10.1161/CIRCRESAHA.122.322244

[190] M. K. Wang , C. C. Gao , and Y. G. Yang , “Emerging Roles of RNA Methylation in Development, ” Accounts of Chemical Research 56, no. 23 (2023): 3417–3427. 37965760 10.1021/acs.accounts.3c00448

[191] L. Li , X. Lu , and J. Dean , “The Maternal to Zygotic Transition in Mammals, ” Molecular Aspects of Medicine 34, no. 5 (2013): 919–938. 23352575 10.1016/j.mam.2013.01.003 PMC3669654

[192] B. S. Zhao , X. Wang , A. V. Beadell , et al., “m6A‐dependent Maternal mRNA Clearance Facilitates Zebrafish Maternal‐to‐zygotic Transition, ” Nature 542, no. 7642 (2017): 475–478. 28192787 10.1038/nature21355 PMC5323276

[193] S. Geula , S. Moshitch‐Moshkovitz , D. Dominissini , et al., “Stem Cells. M6A mRNA Methylation Facilitates Resolution of Naïve Pluripotency Toward Differentiation, ” Science 347, no. 6225 (2015): 1002–1006. 25569111 10.1126/science.1261417

[194] R. Hauenschild , L. Tserovski , K. Schmid , et al., “The Reverse Transcription Signature of N‐1‐methyladenosine in RNA‐Seq Is Sequence Dependent, ” Nucleic Acids Res. 43, no. 20 (2015): 9950–9964. 26365242 10.1093/nar/gkv895 PMC4787781

[195] Y. Yang , L. Wang , X. Han , et al., “RNA 5‐Methylcytosine Facilitates the Maternal‐to‐Zygotic Transition by Preventing Maternal mRNA Decay, ” Molecular Cell 75, no. 6 (2019): 1188–1202. 31399345 10.1016/j.molcel.2019.06.033

[196] J. Liu , H. Zuo , Z. Wang , et al., “The m6A Reader YTHDC1 Regulates Muscle Stem Cell Proliferation via PI4K–Akt–mTOR Signalling, ” Cell Proliferation 56, no. 8 (2023): e13410. 36722312 10.1111/cpr.13410 PMC10392063

[197] W. Wang , E. Li , J. Zou , et al., “Ubiquitin Ligase RBX2/SAG Regulates Mitochondrial Ubiquitination and Mitophagy, ” Circulation Research Published online July 19, 2024. 10.1161/CIRCRESAHA.124.324285 PMC11264309 38873758

[198] V. A. Golubeva , A. S. Das , C. P. Rabolli , L. E. Dorn , J. H. van Berlo , and F. Accornero , “YTHDF1 is Pivotal for Maintenance of Cardiac Homeostasis, ” Journal of Molecular and Cellular Cardiology 193 (2024): 25–35. 38768805 10.1016/j.yjmcc.2024.05.008 PMC11983483

[199] D. Han and M. M. Xu , “RNA Modification in the Immune System, ” Annual Review of Immunology 41 (2023): 73–98. 10.1146/annurev-immunol-101921-045401 37126422

[200] K. Karikó , M. Buckstein , H. Ni , and D. Weissman , “Suppression of RNA Recognition by Toll‐Like Receptors: The Impact of Nucleoside Modification and the Evolutionary Origin of RNA, ” Immunity 23, no. 2 (2005): 165–175. 16111635 10.1016/j.immuni.2005.06.008

[201] Y. Zhang , W. Hu , and H. B. Li , “RNA Modification‐mediated Translational Control in Immune Cells, ” RNA Biol 20, no. 1 (2023): 603–613. 37584554 10.1080/15476286.2023.2246256 PMC10435004

[202] L. Cui , R. Ma , J. Cai , et al., “RNA Modifications: Importance in Immune Cell Biology and Related Diseases, ” Signal Transduct Target Ther 7, no. 1 (2022): 334. 36138023 10.1038/s41392-022-01175-9 PMC9499983

[203] Y. Liu , Y. Chen , M. Cai , Y. Hong , X. Wu , and S. Li , “m5C methylation Modification Guides the Prognostic Value and Immune Landscapes in Acute Myeloid Leukemia, ” Aging 15, no. 18 (2023): 9858–9876. 37751592 10.18632/aging.205059 PMC10564437

[204] W. Zhao , Y. Wu , F. Zhao , et al., “Scoring Model Based on the Signature of Non‐m6A‐related Neoantigen‐coding lncRNAs Assists in Immune Microenvironment Analysis and TCR‐neoantigen Pair Selection in Gliomas, ” Journal of translational medicine 20, no. 1 (2022): 494. 36309750 10.1186/s12967-022-03713-z PMC9617417

[205] Z. Yu , Q. He , and G. Xu , “Effect of N6‐methyladenosine (m6A) Regulator‐related Immunogenes on the Prognosis and Immune Microenvironment of Breast Cancer, ” Transl Cancer Res 11, no. 12 (2022): 4303–4314. 36644186 10.21037/tcr-22-1335 PMC9834601

[206] Y. Wang , S. Zhang , N. Kang , et al., “Progressive Polyadenylation and m6A Modification of Ighg1 mRNA Maintain IgG1 Antibody Homeostasis in Antibody‐secreting Cells, ” Immunity 57, no. 11 (2024): 2547–2564.e12. 39476842 10.1016/j.immuni.2024.10.004

[207] Y. Yang , M. Li , L. Ding , et al., “EZH2 promotes B‐cell Autoimmunity in Primary Sjogren's Syndrome via METTL3‐mediated m6A Modification, ” Journal of Autoimmunity 149 (2024): 103341. 39577129 10.1016/j.jaut.2024.103341

